# CD44 and its implication in neoplastic diseases

**DOI:** 10.1002/mco2.554

**Published:** 2024-05-23

**Authors:** Yiming Xu, Ziyi Bai, Tianxia Lan, Chenying Fu, Ping Cheng

**Affiliations:** ^1^ Department of Biotherapy Laboratory of Aging Research and Cancer Drug Target, State Key Laboratory of Biotherapy, National Clinical Research Center for Geriatrics, West China Hospital, Sichuan University Chengdu Sichuan China; ^2^ Laboratory of Aging and Geriatric Medicine, National Clinical Research Center for Geriatrics, West China Hospital, Sichuan University Chengdu Sichuan China; ^3^ Department of Biotherapy, Cancer Center, State Key Laboratory of Biotherapy, West China Hospital, Sichuan University Chengdu China

**Keywords:** CD44, neoplastic diseases, signaling pathways, therapeutic strategies

## Abstract

CD44, a nonkinase single span transmembrane glycoprotein, is a major cell surface receptor for many other extracellular matrix components as well as classic markers of cancer stem cells and immune cells. Through alternative splicing of *CD44* gene, CD44 is divided into two isoforms, the standard isoform of CD44 (CD44s) and the variant isoform of CD44 (CD44v). Different isoforms of CD44 participate in regulating various signaling pathways, modulating cancer proliferation, invasion, metastasis, and drug resistance, with its aberrant expression and dysregulation contributing to tumor initiation and progression. However, CD44s and CD44v play overlapping or contradictory roles in tumor initiation and progression, which is not fully understood. Herein, we discuss the present understanding of the functional and structural roles of CD44 in the pathogenic mechanism of multiple cancers. The regulation functions of CD44 in cancers‐associated signaling pathways is summarized. Moreover, we provide an overview of the anticancer therapeutic strategies that targeting CD44 and preclinical and clinical trials evaluating the pharmacokinetics, efficacy, and drug‐related toxicity about CD44‐targeted therapies. This review provides up‐to‐date information about the roles of CD44 in neoplastic diseases, which may open new perspectives in the field of cancer treatment through targeting CD44.

## Graphical abstract

The expression of CD44 is associated with stemness in cancer cells and activation in immune cells. In this review, we have mainly discussed the influence of CD44 expressing in cancer cells and therapeutic strategies through targeting CD44.

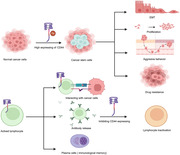

## 1 INTRODUCTION

CD44, a nonkinase transmembrane glycoprotein,^1^ is expressed in various human cell types, such as immune cells, differentiated cells, cancer cells, and so on.^2^, ^3^ CD44 has numerous ligands, such as hyaluronic acid (HA),^4^ osteopontin (OPN),^5^ and serglycin.^6^ The extracellular region of CD44 binding to ligands has been found to involve various of signaling pathways associated with physiological and pathological processes,^7^, ^8^ in particular, pathways related to carcinogenesis and tumor progression including proliferation and migration of cells, drug resistance, as well as epithelial–mesenchymal transition (EMT).^9^, ^10^, ^11^

Alternative splicing of the CD44 gene produces two splice isoforms, CD44s and CD44v. Notably, CD44s and CD44v play an overlapping or distinct role in cancers. In most cases, CD44s is associated with tumor growth^12^ and progression,^13^ while CD44v, such as CD44v3 and CD44v6, is associated with invasiveness^14^ and chemoresistance.^15^

Accumulating evidence indicates that CD44 can regulate numerous cancer‐associated signaling pathways to influence cancer cell motility, EMT, and stemness.^16^, ^17^, ^18^ In recent years, a number of novel functions of CD44 and its associations with cancers have been revealed. Recent advances in understanding the complex interactions of CD44 with ligands have led to the development of CD44 not only as an important cancer stem cells (CSCs) marker but also as a potential cancer therapeutic target.^3^, ^19^, ^20^ In neoplastic diseases therapeutic areas, it is very crucial to clarify the mechanism of CD44 in the signaling pathways, thereby delaying, treating, and preventing the development of tumors through targeting CD44. However, there are relatively few clinical studies about CD44‐targeted therapies in cancer treatment. Hence, an updated and comprehensive understanding of CD44 is very crucial, which contributes to the research and development of innovative CD44‐targeted therapeutic strategies.

In this review, we discuss the structure and ligands of CD44 briefly. And we focus on the biological functions of CD44 in different cancers and cancer‐related signaling pathways regulated by CD44 and provide critical assessment of therapeutic strategies and clinical studies through targeting CD44.

## 2 THE STRUCTURE OF CD44

**FIGURE 1:**
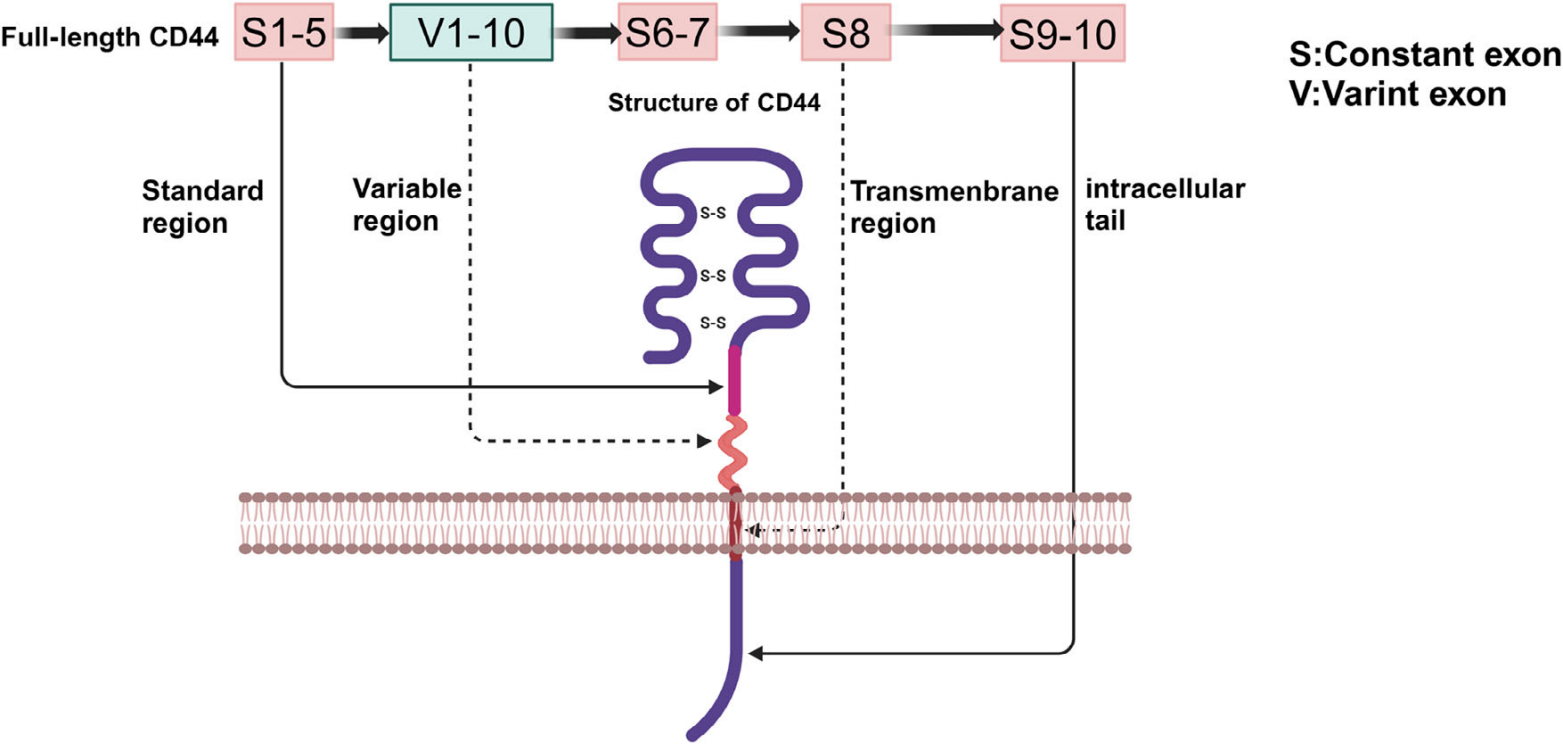
The gene arrangement and structure of CD44 in mice. The full‐length CD44 in mice has 20 exons, including constant exons and variant exons. The exon of S1–5, V1–10, S8, S9–10 corresponding to the standard region, variable region, transmembrane region and intracellular tail of CD44 respectively. This scheme was generated using Biorender.

CD44 is a nonkinase cell surface transmembrane proteoglycan that includes an ectodomain, a stem region, composed of standard region and variable region, a transmembrane region and an intracellular tail.^21^ In human, the gene encoding CD44 protein is on chromosome 11, while in mice, the gene is on chromosome 2, which comprises 19 exons and 20 exons.^22^ Comparing with mice, CD44v1, includes homolog exon 6, is not expressed in humans.^23^ For the reason that the variable exon 6 of the human gene has a stop codon that is normally not expressed in human *CD44*,^24^ CD44s is the most common and smallest CD44 isoform, which includes the constant exons 1−5 and 16−20. Based on the structure of CD44s, CD44v consists of variable exons 6−15 located in the exon 1−5 and 16−20 regions by alternate splicing or insertion.^25^ CD44v1–v10 correspond to alternative splicing or insertion of variable exons 6–15, respectively,^26^ which have different functions in neoplastic diseases.^27^ Moreover, a CD44v isoform could have more than one variable exon. For example, CD44v8–10 is based on CD44s structure with the insertion of v8, v9, and v10, three variable exons.^28^ The correspondence between structure and gene arrangement of CD44 in mice is shown in Figure 1.

## 3 CD44 IN NEOPLASTIC DISEASES

**FIGURE 2:**
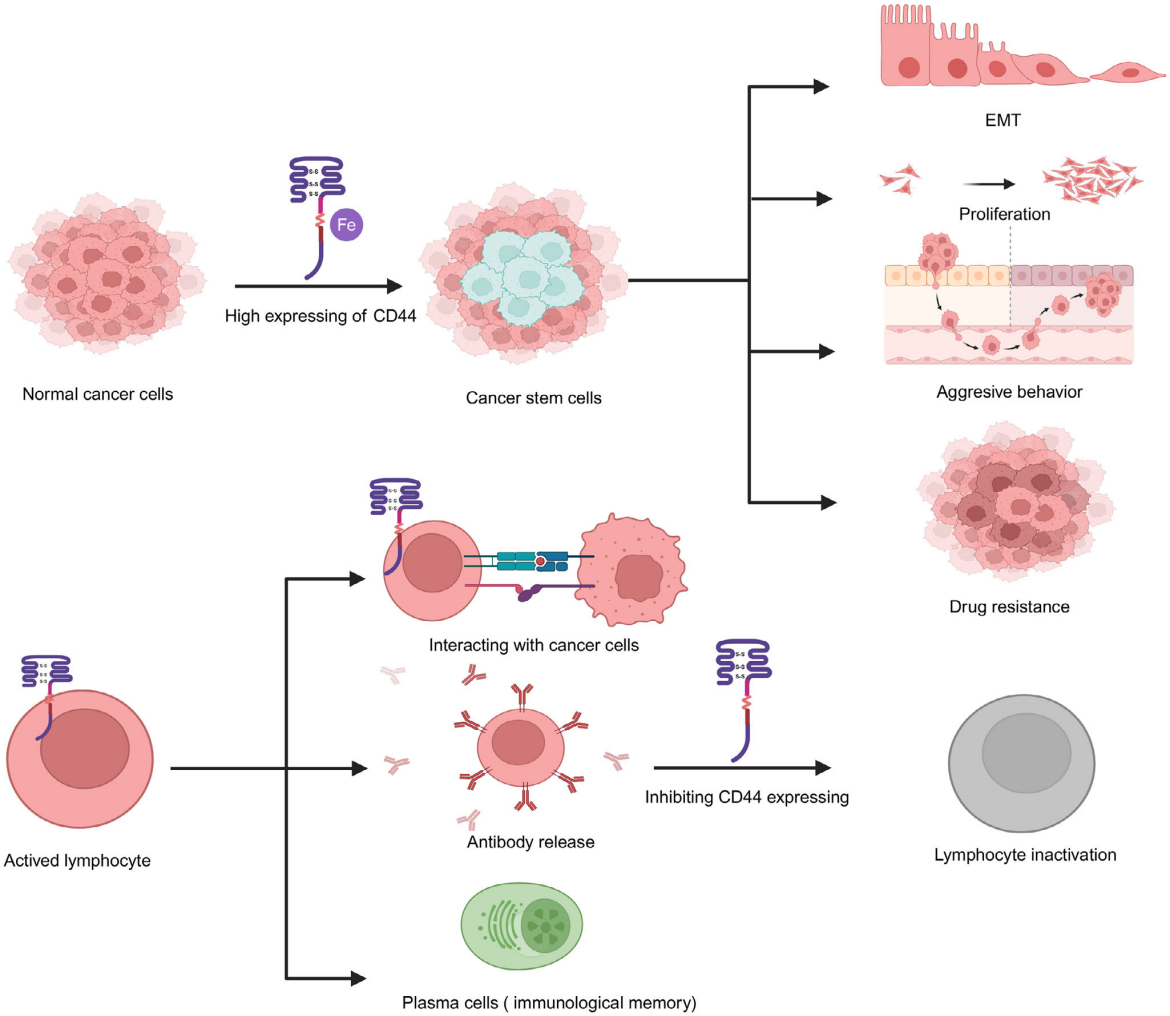
The role of CD44 expressing cancer stem cells and immune cells in neoplastic disease. The expression of CD44 on cancer cells contributes to stemness mediated by iron, which is associated with EMT, proliferation, aggressive behavior, and drug resistance. On top of that, CD44 expressing immune cells is associated with adhesion, migration, and the activation of immune cells and immunological memory. Inhabiting the expression of CD44 could cause immune cells inactivation and thus tumor immune tolerance. This scheme was generated using Biorender.

CD44 has been reported as a classic marker of CSCs, which is mainly associated with of iron endocytosis‐mediated cellular plasticity.^29^ A recent study has found that in cancer cell lines, through the endocytosis of iron‐bound HA regulated by CD44, the EMT was enhanced.^30^ The effects of the expressing of CD44 on immune cells and cancer cells in neoplastic diseases are summarized in Figure 2. Moreover, previous studies have found that the expression of CD44 isoforms in tumors plays crucial roles.^31^ Some researchers have demonstrated that part of CD44v is associated with aggressive tumor progression and drug resistance,^32^ whereas CD44s is involved in tumorigenesis and tumor growth.^13^ However, recent studies have shown the inverse result. For instance, CD44s has been shown to promote migratory, invasive, and lung metastatic potential in breast cancer.^33^ In 3D cultures, CD44 switching from the standard isoform to the variant 6 isoform was found to be associated with EMT in gastric cells.^34^ The different or overlapping functions of CD44s and CD44v may depend on the difference in tumor types. In addition, different induction conditions, accompanied by alternative splicing of CD44 isoforms, may lead to different expression of features in tumor cells. The correlation between different cancers and CD44 are summarized in Table 1. Moreover, CD44 could also play its biological roles by interacting with ligands and messenger molecules, such as HA,^35^ OPN,^36^ chondroitin sulfate (CS)^37^ and growth factors^38^ mainly found in the tumor microenvironment. In this part, we have not only made a discussion in terms of different kinds of cancers but also discussed the effects of CD44 interacting with its different ligands on cancers.

**TABLE 1:** The correlation between different cancers and CD44.

Cancer type	Role of CD44 in cancer progress	References
Brain cancer	Poor prognosis, tumor progression, aggressive behavior	^39^, ^40^, ^41^
Head and neck cancer	Stemness, tumor progression	^48^, ^49^
Breast cancer	Aggressive behavior, tumor progression, stemness, poor prognosis	^56^, ^57^, ^58^, ^59^, ^60^
Kidney cancer	Poor prognosis, aggressive behavior, tumor growth	^65^, ^66^, ^67^
Liver cancer	Cancer initiation, poor prognosis, stemness	^72^, ^73^, ^74^
Pancreatic cancer	Tumorigenicity, clinicopathological features, aggressive behavior	^80^, ^81^, ^82^
Gallbladder cancer	Stemness, aggressive behavior, tumor progression, poor prognosis	^91^, ^92^, ^93^, ^94^
Esophageal cancer	Tumor progression, stemness, poor prognosis, aggressive behavior	^97^, ^98^, ^99^, ^101^, ^102^
Prostate cancer	Tumor progression, stemness, aggressive behavior	^104^, ^105^, ^106^, ^109^
Gastrointestinal cancer	Tumor progression, stemness, aggressive behavior, CSCs self‐renewal characteristic tumorigenesis	^114^, ^115^, ^116^, ^117^, ^119^
Melanoma	Stemness, tumorigenesis, aggressive behavior	^122^, ^123^, ^125^
Squamous cell carcinoma	Aggressive behavior, stemness, tumorigenesis, tumor progression	^128^, ^129^, ^130^
Sarcoma	Tumor progression, stemness, aggressive behavior, poor prognosis	^133^, ^134^, ^135^, ^138^, ^139^

### 3.1 Brain cancer

It has been shown that overexpression of CD44 is linked to poor prognosis,^39^ tumor progression,^40^ and aggressive behavior^41^ of tumors. For instance, CD44 is a possible CSCs marker in meningioma; the high expression of CD44 was associated with a shorter progression‐free survival.^42^ Furthermore, in meningioma, the level of CD44 expression is correlated with the grade of the tumor and its invasiveness.^43^ Moreover, inhibiting CD44 dimerization via verbascoside has been reported to suppress stem cell‐like cell properties and tumor cell growth in glioblastoma.^44^ Recent studies have found that tumor cells with high expression of CD44 is associated with brain metastases. It has been revealed that in lung, melanoma, and breast cancer patients, the circulating tumor cells expressing CD44 was a prognostic marker for brain metastases.^45^ Moreover, in lung adenocarcinoma, GPR124‐enhanced trans‐endothelial migration mediated brain metastases caused by lung CSCs with high expression of CD44.^46^ Furthermore, in breast cancer brain metastases patients, a retrospective transversal study revealed that CD44 was associated with worse overall survival.^47^ In general, CD44 is a promising marker associated with brain metastases in cancer patients, which provides us with the new insight into stratification of patients and therapy clinically.

### 3.2 Head and neck cancer

It is found that CD44 seems to be a classic CSCs marker,^48^ which is also associated with tumorigenesis in head and neck cancer.^49^ In head and neck squamous cell carcinoma (HNSCC) CSCs mouse models, targeting CD44 was shown to inhibit PI3K–4EBP1–SOX2 signaling and tumor growth and decrease the number of CSCs.^50^ Moreover, it is also suggested that the switch from CD44s to CD44v8–10 was associated with tumorigenic phenotypes.^52^ In addition, CD44^+^ cells were reported to stimulate tumor angiogenesis in HNSCC.^53^ In the invasion zone of HNSCC, Odenthal et al.^54^ found that CD44v6 could express constitutively. This suggests that targeting CD44v6 could be used to trustworthy near‐infrared detection. Furthermore, Choi et al.^55^ has found that the interaction of COL1A1 and CD44 between fibroblasts and malignant cells was associated with HNSCC progression. Therefore, CD44 could be a promising biomarker for clinical diagnosis, and targeting CD44 could be a treatment strategy for drug resistance and tumor metastasis.

### 3.3 Breast cancer

In breast cancer, CD44 plays key roles in aggressive tumor behavior,^56^ tumor progression,^57^ CSCs trait induction,^58^, ^59^ and prognosis.^60^ Rokana et al.^51^ found that aggregated tumor cells with highly expressed CD44 could promote tumorigenesis and polyclonal metastasis. Moreover, depletion of CD44 has been found to effectively prevent the aggregation of tumor cell and decrease the levels of PAK2.^51^ Interestingly, CD44s was reported to inhibit breast cancer stemness, while the cleaved product of CD44 was reported to contribute to breast cancer stemness.^61^ However, bioinformatics analysis of breast cancer patients showed the reverse result. Notably, multiple studies have reported inconsistent results. For instance, CD44s being positively associated with CSCs gene signatures, while CD44v exhibited an inverse association.^62^ Compelling evidence further suggests that the splicing switch of CD44 may play inverse roles in breast cancer. For instance, Yang et al.^63^ found that enhanced transformation of CD44^high^ to CD44^low^ cancer cells induced migration and invasion behavior. Additionally, CD44 isoform switching from CD44s to CD44v resulted in an increase in the stemness of triple‐negative breast cancer.^64^ Targeting different CD44 isoforms or inducing CD44 alternative splicing may be a favorable treatment strategy for breast cancer. The specific effects of CD44v and CD44s need to be further studied.

### 3.4 Kidney cancer

It is well documented that CD44 could serve as biomarkers that reflect poor prognosis^65^ and cancer risk in kidney cancer.^66^, ^67^ In transformed cells within multilayered epithelia, CD44 and collagen XVII were reported to play a key role in the clonal expansion.^68^ Moreover, the ferroptosis‐related gene CHAC1 was shown to contribute to poor prognosis in kidney renal clear cell carcinoma associated with the expression of the checkpoint gene CD44.^69^ Notably, it was reported that silencing CD44, PLOD1, and PLOD2 genes could inhibit the proliferative and invasive potential of renal cancer cells, which suggested that these genes may serve as renal cell carcinoma oncogenes.^70^ Interestingly, unlike the role of CD44 in other cancer, it was found to display the opposite effect. In renal carcinoma, CD44^−^ cancer cells display stem‐like properties and show a higher level of invasiveness.^71^ All the above results show that targeting CD44 or inhabiting CD44 expression could be a promising therapeutic strategy to suppress kidney progression.

### 3.5 Liver cancer

CD44 is clearly associated with liver cancer initiation,^72^ poor prognosis,^73^ and cancer cells stemness.^74^ CD44 is expressed in carcinogen‐exposed hepatocytes in a STAT3‐dependent manner.^75^ Accordingly, CD44v6 was also found to be a promising biomarker. In patients with grade 1 intrahepatic carcinomas and grade 1 hepatocellular carcinomas (HCCs), TIPRL/LC3/CD133/CD44 also play a key role in prognosis.^76^ Furthermore, compelling evidence suggests that CD44 is related to stemness in liver cancer. CD44‐positive HCC patient‐derived organoids were shown to be obviously resistant to sorafenib via Hedgehog signaling.^77^ Besides, in CD24^+^/CD44^+^ cells, knocking down the signature gene CTSE was found to significantly inhibit the self‐renewal potential of HCC cells.^78^ CD44 surface markers were found in cancer cells with stemness properties, which indicate that, in HCC, targeting CD44 expressed in tumorigenic cells through JAK/STAT pathway is a promising therapeutic strategy^79^

### 3.6 Pancreatic cancer

In pancreatic cancer, CD44 is involved in tumorigenicity,^80^ clinicopathological features,^81^ and invasiveness.^82^ In pancreatic ductal adenocarcinoma (PDAC) patients with liver metastasis or poor prognosis, the expression of CD44v6 and complement C1q binding protein was higher.^83^ Moreover, CD44^+^ PDAC cells were reduced with nimbolide treatment.^84^ A meta‐analysis showed that CD44 overexpression contribute to a poor 5‐year overall survival rate and lymph node invasion.^85^ On top of that, CD44^+^ stem cells from the Panc‐1 cell line have been found to be involved in multiresistance and metastasis.^86^ In addition, more recent research has demonstrated that the interaction of HA and CD44 could be used for drug delivery in pancreatic cancer. HA‐based carriers have been shown to target tumors via interaction with CD44 in pancreatic cell lines.^87^ HA‐based nanomicelles loaded with 3,4‐difluorobenzylidene curcumin could also kill CD44^+^ stem‐like pancreatic cancer cells.^88^ Additionally, HA‐conjugated polyamidoamine dendrimers were revealed to deliver 3,4‐difluorobenzylidene curcumin to pancreatic cancer cells overexpressed CD44.^89^ Furthermore, Kang et al.^90^ reported a nanoparticle loaded with anticancer drug consists of HA, which could target CD44 in pancreatic cancer cells to eliminating tumor‐resident intracellular bacteria. Hence, targeting CD44 drug delivery systems in cancer cells provide an avenue for pancreatic cancer treatment. Moreover, decreasing the expression of CD44 in pancreatic cancer is also a promising therapeutic strategy.

### 3.7 Gallbladder cancer

There are few studies on CD44 in gallbladder cancer (GBC), which mainly focus on the different functions of CD44 isoforms and the stemness of cancer cells expressing CD44.^91^, ^92^ CD44v9 and CD44s cells were found to play key parts in the progression and metastasis of GBC respectively, and the isoform switch triggered EMT.^93^ Interestingly, a research suggested that CD44v8–10 expression was closely associated with perineural invasion, venous invasion, and lymph node metastasis, and in the clinic, the patients with CD44v8–10^+^ tumors showed poor prognosis.^94^ In addition, the high expression of CD44 as a stem cell marker in sphere clones of the human GBC cell line GBC‐SD was further explored.^95^ Interestingly, in general, the stemness of cancer cells is positively correlated with drug resistance, but the opposite effect is shown in GBC.^96^ All the studies indicate that the characteristics of CD44 in GBC provide some new thought in diagnosis and treatment of GBC.

### 3.8 Esophageal cancer

High expression of CD44 plays roles in tumor progression and stemness in esophageal squamous cell carcinoma (ESCC).^97^, ^98^, ^99^ CD44 is a novel stem cell marker in ESCC that has been reported to be eliminated by inhibiting the canonical NOTCH pathway.^100^ Moreover, CD44v9 was shown to be strongly associated with EMT and poor prognosis in patients with ESCC.^101^ Furthermore, microRNA (miR)‐34a suppressed invasion and metastasis in ESCC by regulating CD44.^102^ In addition, the latest research has found that the alternative splicing of CD44 isoform from CD44s to CD44v8–10 is associated with ESCC metastasis and poor prognosis in clinic.^103^ The CD44v isoforms has similar functions in ESCC, which provide useful insights for the development of ESCC treatment and prognostic biomarkers.

### 3.9 Prostate cancer

CD44 is involved in prostate cancer, and different CD44 isoforms play different roles in tumor progression and stemness.^104^, ^105^, ^106^ A recent study showed that TGF‐β1‐mediated alternative splicing could switch CD44v to CD44s, which enhanced EMT and stemness in human prostate cancer cells.^107^ In DU145 and PC3 prostate cancer cells, the high expression of CD44v4, v5, and v7 mediated by sulforaphane contributed to tumor cell growth and proliferative activity.^108^ Furthermore, CD44^high^ stem cell‐like cells have been found to be involved in drug resistance and invasive phenotypes in DU145 and PC3 cell populations.^109^ In addition, high expression of CD44 was reported to be associated with prostate cancer cell migration and proliferation.^110^ Accumulating evidence suggests that CD44 is a stem cell marker in prostate cancer. Translationally controlled tumor protein (TCTP) was closely associated with survival factor of stem cells, and the TCTP inhibitor sertraline highly downregulated the expression of CD44.^111^ Enzalutamide has been found to induce stem‐like characteristics to acquire resistance, and CD44 has been found in enzalutamide‐resistant cells.^112^ Additionally, STAT3 also contributes to the cell stemness and activation of the CSCs marker CD44 in PC cells.^113^ In general, CD44v isoform is associated with cancer stemness. However, only some studies have identified the specific CD44v isoform. The specific CD44v isoforms in other studies are unclear, which need further study.

### 3.10 Gastrointestinal cancer

Abundant evidence indicates that CD44 contributes to tumor progression^114^ and stemness^115^ in gastrointestinal cancer. It was revealed that MUC5AC interacting with CD44 promoted cell invasive and migrative potential and decreased apoptosis of colorectal cancer (CRC) cells via Src signaling.^116^ In addition, PD‐L1 was found to increase the population sizes and the tumorspheres forming ability in CD133^+^CD44^+^ cell, which resulted in colorectal CSCs self‐renewal.^117^ A previous study revealed that CD44, a stem cell marker in gastric cancer (GC) lines, could be suppressed by DAXX.^118^ Interestingly, CD44 was also found to play either different or overlapping roles in different gastrointestinal cancers, which may be related to the species of CD44 and its isoforms. A previous study revealed that CD44v8–10, but not CD44s, expressing could restore the tumor‐initiating potential of GC cells reduced by silencing total CD44.^119^ Moreover, CD44v8–10 also plays an important part in regulation of ROS defense and tumor growth by p38 (MAPK) and p21 (CIP1/WAF1) in human gastrointestinal cancer cells.^120^ CD44v6 was found to be a stem cell marker, and its overexpression promoted migration and metastasis in colorectal CSCs by activating Wnt/β‐catenin.^14^ In addition, accumulating evidence suggests that miRs binding to CD44 plays key roles in drug resistance, tumor growth, and stemness. For instance, miR‐302a binding to CD44 was shown to suppress CSCs‐like properties and restore cetuximab (CTX) responsiveness in CRC.^121^

### 3.11 Melanoma

CD44 is a CSCs marker^122^ in melanoma cancer and participates in tumor initiation progression.^123^ Wei et al.^124^ found that downregulated RNF128‐activated Wnt signaling induced cellular EMT and stemness by ubiquitinating and degrading CD44/cortactin. Additionally, the depalmitoylation of the prometastatic cell adhesion molecule CD44 was shown to result in increased melanoma invasion via Wnt5a.^125^ Compelling evidence suggests that drugs targeting CD44 in melanoma is a promising strategy for melanoma treatment. For instance, expressing BMP4/7‐dependent Id1/3 protein was reported to decrease the survival rate in melanoma patients promoted by HA–CD44 interactions with BMPR.^126^ Moreover, nanodrug based on CS could target CD44 to treat melanoma by inducing mitochondrial apoptosis.^127^

### 3.12 Squamous cell carcinoma

CD44 plays a role in various squamous cell carcinomas and has been shown to be mainly involved in EMT^128^ and cancer stemness.^129^ In HNSCC, HA binding to CD44 was found to increase CSCs numbers by PI3K–4EBP1–SOX2, whereas CD44 binding to VCAM‐1 contribute to invasiveness by ezrin/PI3K. Moreover, the switch from CD44v8–10 to CD44s was reported to promote EMT and participate in tumor invasion,^52^ which is contrary to the results in ESCC of Yu et al.^102^ In mouse and human squamous cell carcinoma, it has been demonstrated that induction of a hybrid EMT state contributes to tumor initiation, progression, invasiveness, stemness, and metastasis by activating the CAMK2–CD44–SRC axis.^130^ Moreover, in ESCC patients, SOCS6 has been shown to significantly decrease the population of CSCs expressing the surface biomarker CD24^low^/CD44^high^ to overcome radioresistance.^131^ Furthermore, in CD44^high^ OSCC cells, TGF‐β1 was found to induce amoeboid‐to‐mesenchymal transition (AMT) via activating ERK and phosphorylating Cofilin‐1.^132^ In summary, the role of CD44 in squamous cell carcinoma could serve as a vital area for drug development and tumor marker.

### 3.13 Sarcoma

Compelling evidence suggests that the overexpression of CD44 in most sarcomas participates in tumor progression,^133^, ^134^ stemness,^135^ and dissemination.^134^ CD44 was found to increase the resistance of osteosarcoma cells to doxorubicin by upregulating multidrug resistance 1 protein expression.^136^ The human osteosarcoma cell lines MNNG/HOS and 143B were both highly metastatic, and CD44 was reported to be knocked out by CRISPR/Cas9. Additionally, inhibiting cell proliferation and tumor sphere formation cultured in 3D environment was found to depend on CD44 inactivation.^137^ Furthermore, in a mouse fibrosarcoma model, the increased expression of human CD44s was shown to promote micrometastasis events.^138^ Moreover, in osteosarcoma, CD44v6 is an important prognostic factor of patient prognosis.^139^ In sarcomas, research on the roles of CD44 is conductive to understanding the pathogenesis of these rare cancers.

### 3.14 Effects of CD44 interacting with HA on cancers

HA, a linear glycosaminoglycan (GAG),^140^ binds to the N‐terminus of the extracellular domain of all CD44 isoforms.^141^ Aruffo et al.^142^ first proposed the relationship between CD44 and HA. Through endocytosis mediated by iron,^143^, ^144^ the interaction between CD44 and HA plays crucial role in the progression,^145^ invasion,^146^ and chemoresistance^147^ of cancer cells. As reported, patients with CD44s‐positive tumors may gain a survival benefit from HA–irinotecan, which is a formulation of HA and irinotecan.^148^ In tumors with increased expression of CD44, the using of HA‐based nanocarriers was reported to have the benefits in the enhancement of drug delivery, the increase therapeutic efficacy with low cytotoxicity, the inhibition of tumor growth, as well as the high potential for targeted chemotherapy.^149^ Moreover, kynureninase associated with the onset and development of breast cancer was found to be upregulated by CD44, which was induced and activated by HA.^141^ In addition, CD44 isoforms also regulate the uptake and expression of HA. It has been revealed that the expression of cancer cells with CD44v can negatively influence the uptake of HA, whereas cells expressing CD44s has positive correlations with the uptake and expression of HA. Moreover, the ability of HA uptaking varies across the cell lines. CD44s^high^ cancer cells could uptake HA more efficiently compared with CD44s^low^ human dermal fibroblasts.^87^ In summary, the interaction of CD44 with HA and different CD44 isoforms regulates the ability of HA uptaking and expression have effect on cancer, which provides important advances with respect to cancer treatment strategies based on HA and the of selection of different CD44 isoforms as a target.

### 3.15 Effects of CD44 interacting with OPN on cancers

OPN, a phosphorylated glycoprotein, which expressed in the mineralized extracellular matrix (ECM) of bone in normal tissues cells such as fibroblasts, osteoblasts, and osteocytes.^150^, ^151^ As one of the classical and important ligands of CD44, OPN affects the proliferation and invasion of tumor cells as well as inflammation in normal cells, by regulating related signaling pathways.^150^, ^152^ Activation of the JUN N‐terminal kinase pathway has been shown to contribute to the promotion of clonogenicity and tumor growth in a xenograft model via OPN secreted by tumor‐associated macrophages.^153^ In PC3 cells, OPN binds to CD44 receptors that could inhibit the c‐RaF and ERK1/2 by activating the AKT pathway, thereby inhibiting cell cycle arrest.^154^ Additionally, Yang et al.^155^ found that the interaction of CD44 with OPN could promote tumor‐associated mesenchymal stem cells formation, leading to lung cancer cells invasion and migration. Moreover, decreased expression of interferon regulatory factor 8 in colon carcinoma was reported to increase the expression of OPN, which is associated with lower patient survival rate. This phenomenon is associated with the interaction of OPN with CD44 leading to immune escape.^156^ Taking together, high expression of OPN could promote immune escape of tumor cells, which may be related to regulate T cells activation through OPN interacting with its receptor CD44 and compensating for the function of PD‐L1.^157^

### 3.16 Effects of CD44 interacting with serglycin on cancers

Serglycin has been shown to be a ligand for CD44. Previous studies have found that the binding of CD44 to serglycin is implicated in tumorigenesis and prognosis.^158^ The attachment of GAGs to serglycin could facilitate this binding.^159^ For instance, the proteoglycan serglycin was reported to promote the migration of non‐small cell lung cancer cells via the binding of its GAG motif to CD44.^160^ Moreover, serglycin binding to CD44 was shown to regulate the expression of CD44 in a β catenin‐dependent manner, a finding that might improve the poor prognosis in triple‐negative breast cancer.^161^ In addition, the interaction of serglycin with CD44 was revealed to promote tumorigenesis in giant cell tumors of bone via activating focal adhesion kinase.^162^ There are few studies on serglycin and CD44 in tumors, but for the important role of serglycin binding to CD44 in tumorigenesis, it is believed that there will be more therapeutic strategies based on the regulating the binding of the two in future studies.

### 3.17 Effects of CD44 interacting with CS on cancers

In recent years, the studies on CD44 and CS mainly focused on the nanodelivery systems based on the specific binding between CD44 and CS.^163^ Accumulating evidence suggests that CS modifies the drug delivery of nanoparticles that target CD44 into tumor cells with low cytotoxicity.^164^ Nanoparticles composed of CS, doxorubicin, and bovine serum albumin targeting CD44 were reported to suppress 4T1 tumor growth.^165^ Additionally, a nanosystem targeting CD44, composed of d‐α‐tocopherol polyethylene 1000 glycol succinate and CS dual‐modified lipid‐albumin, was found to deliver paclitaxel into multidrug‐resistant tumor cells, thereby overcoming drug resistance.^166^ Moreover, Chu et al.^167^ found that suppressing the expression of CD44 and integrin β1 could reduce the invasiveness of glioma cells by suppressing CS synthase 1. All the evidence shows that CD44 plays an important role in cancer treatments by nanoparticles modified by CS and other drugs.

### 3.18 Effects of CD44 interacting with matrix metalloproteinases on cancers

Matrix metalloproteinases (MMPs) is a large family of zinc endopeptidases whose members can interact with CD44. The binding of CD44 to MMPs has also been reported to mediate tumor growth, stemness, as well as the aggressive behavior.^168^, ^169^ It has been shown that hybrid nanoparticles targeting CD44 or MMPs have antitumor efficacy in 4T1 breast tumor,^170^ MCF‐7, and MDA‐MB‐231 cells^171^ by inhibiting the expression and activity of MMPs. Also, polyphenols extracted from Artemisia annua L. showed anticancer effects by suppressing CD44 and MMP9 in radio‐resistant MDA‐MB‐231 human breast cancer cells.^172^ Interestingly, CD44 has been shown to be both positively and negatively correlated with the expression of MMPs.^173^ For instance, the overexpression of CD44 was reported to reduce the levels of MMPs for the maintenance of ECM homeostasis.^174^ In Papadopoulou's study,^175^ the increase in MMP1 and MMP13 expression followed by the decreased expression levels of CD44 was found to promote tumor growth. Another study also showed that MMP2 activation contributed to decrease the number of CD44^+^/CD24^−^ cells in MDA‐MB‐231 cells after normothermic microwave treatment.^176^ In contrast, inhibiting the expression of MMPs also inhibited the expression of CD44, for instance, DSPP/MMP20 gene silencing resulted in downregulation of CD44, a marker of oral squamous cell carcinoma (OSCC), and increased sensitivity to cisplatin.^177^ Moreover, CD44 was shown to promote lung ADC cell invasion by regulating MMP‐2 expression.^178^ Also, in PC3 cells and breast carcinoma cells, the intracellular domain of CD44 was found to form a complex with the RUNX2 protein, which mediated cancer metastasis, migration, and progression through activation of MMP‐9.^179^ In encapsulated papillary carcinoma (EPC), the high expression of CD44s induced high expression of MMP2 associated with invasion.^180^ Overall, the complex relationship between CD44 and MMPs expression provides us with more ideas for selecting different targets in cancer therapeutic strategies.

### 3.19 Effects of CD44 interacting with fibronectin on cancers

It was revealed that fibronectin (FN) in the ECM could interact with CD44, which is involved in cell metastasis,^181^ migration,^182^ adhesion,^183^ and survival.^184^ In endothelial cells, space microgravity was found to modulate the expression of CD44 and restrain collagen I and FN deposition, which involved cellular adhesion.^185^ Additionally, by using a CD44 antibody to partially inhibit extracellular FN signaling, a significantly decrease in adhesion and survival of melanoma cells was obversed.^186^ Furthermore, FN type III domain‐containing protein 3B circular RNA increased migration and invasion in GC with a decline in CD44.^187^ Although there are few studies on regulating the interaction of CD44 with FN to treat cancer, owing to the key role of FN in cell migration and adhesion, this aspect may be a promising therapeutic strategy in cancer metastasis.

The relationship in neoplastic diseases between CD44 and its ligands is complex, and the expression of the two may be positively or negatively correlated. The main studies is about CD44 rather than a specific CD44 isoform interacts with its ligands. Together, CD44 interacts with its ligands plays key roles in cancer progression. All the abovementioned studies provide new insights into cancer therapy by interfering the interaction of CD44 with its ligands.

## 4 CANCER‐ASSOCIATED PATHWAYS REGULATED BY CD44

**FIGURE 3:**
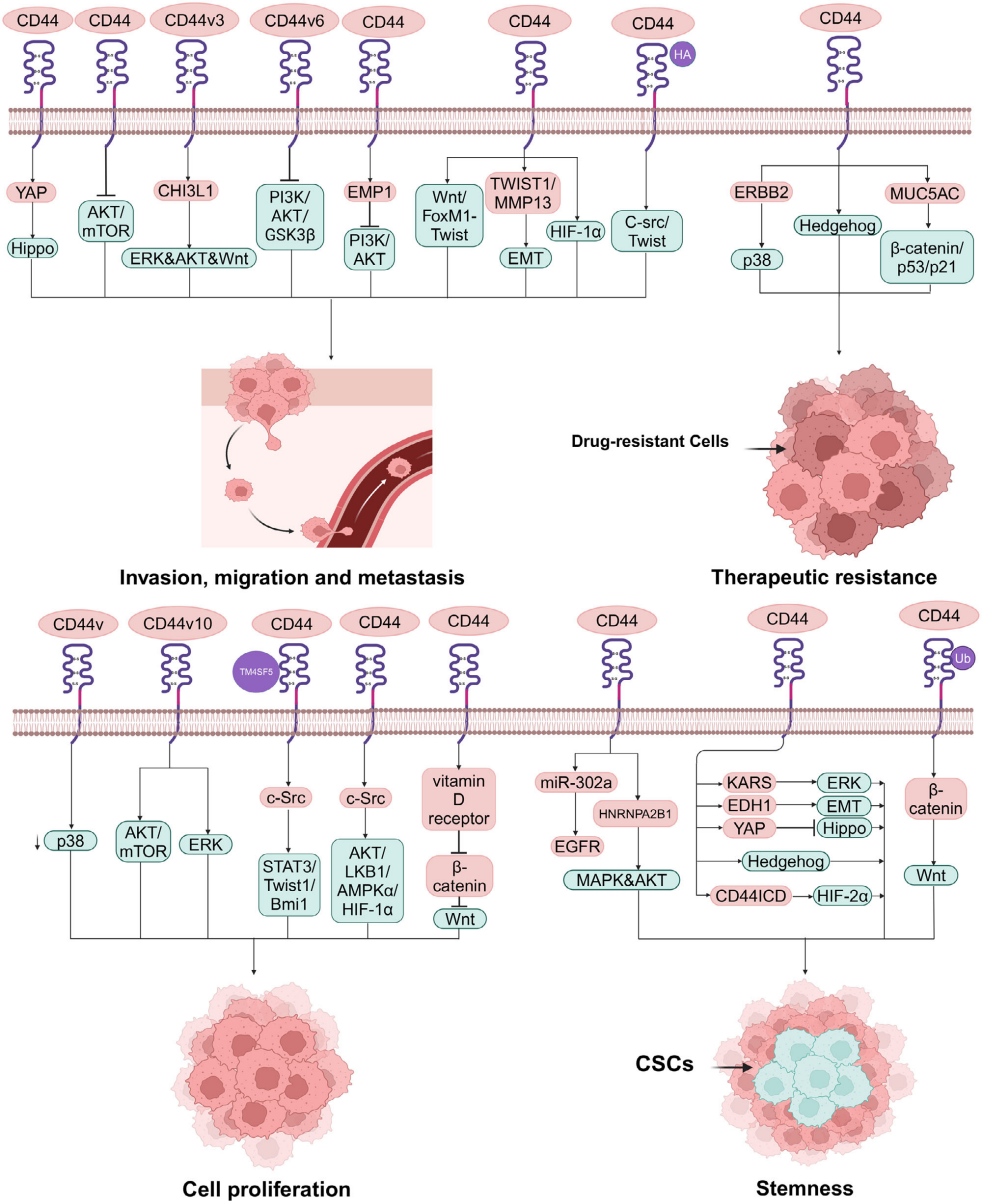
CD44‐mediated cancer‐associated signaling pathways. CD44 can contribute to cancer invasion, migration, and metastasis through Hippo–YAP, HIF‐1α, and Wnt–FoxM1–Twist signaling. CD44v3 binds to CHI3L1, resulting in metastasis of GC via the ERK–AKT–Wnt pathway. Silencing of CD44v6 blocks PI3K/AKT/GSK3β signaling pathway, EMP1‐affected CD44 expression inhibits the PI3K/AKT signaling pathways, CD44 inhabits the phosphorylation of AKT/mTOR, HA‐induced CD44 interaction with C‐Src‐activated Twist can result in inhibiting cancer aggressive behavior. The TWIST1–CD44–MMP13 axis involves in tumor aggressiveness through EMT. MiR‐302a binds to CD44, resulting in suppressing CSCs‐like properties via EGFR‐mediated MAPK and AKT signaling. CD44^high^ CSCs activate the AKT and MAPK pathways via the expression of HNRNPA2B1, CD44 activates KRAS/ERK signaling, EDH1 interaction with CD44 inhabiting Hippo pathway, the upregulation of CD44 activates YAP through inactivation of Hippo signaling pathway, CD44ICD binding to HIF‐2α via activation of HIF‐targeting genes, and the ubiquitination and degradation of CD44/cortactin via activating Wnt/β‐catenin signaling can result in cancer stemness and EMT features. Ablation of CD44v via activation of p38 signaling and Vitamin D receptor‐induced inhibition of CD44 via Wnt/β‐catenin signaling can result in inhabiting tumor growth and cell proliferation. CD44v10 activates ERK/p38 MAPK and AKT/mTOR signaling, the TM4SF5/CD44 interaction via activating c‐Src/STAT3/Twist1/Bmi1 signaling can result in facilitating cell proliferation. Reducing the glycolytic phenotype of cancer cells through the c‐Src/AKT/LKB1/AMPKα/HIF‐1α signaling pathway can silence CD44 to regulate cell proliferation. CD44 collaborates with ERBB2 to promote the radioresistance of cancer cells via p38 phosphorylation. CD44^+^ cancer cells reverse sorafenib resistance via suppressing Hedgehog signaling. Upregulation of CD44 confers chemoresistance via β‐catenin/p53/p21, which is associated with the secretory mucin MUC5AC. This scheme was generated using Biorender.

Activation and deactivation of CD44 isoforms regulate the activities of the components of signaling pathways, including enzymes,^188^ protein kinase pathways,^189^, ^190^ and transcription factors,^191^ which have been found to be associated with tumor initiation progression and aggressive behavior.^192^, ^193^ Moreover, CD44 is also found to be a component in some cancer‐associated signaling pathways.^194^ Some signaling pathways associated other cancers regulated by CD44 are summarized in Figure 3. Moreover, the biological functions of different CD44 isoforms in cancer are summarized in Table 2

**TABLE 2:** Biological functions of different CD44 isoforms in cancer.

CD44 isoforms	Biological functions	References
CD44s	Tumorigenesis, tumor growth, aggressive behavior, micrometastasis events, inhabiting stemness, CSCs characteristics, EMT	^13^, ^52^, ^61^, ^62^, ^93^, ^107^, ^138^, ^180^
CD44v	Invasiveness, stemness, tumor growth	^62^, ^64^, ^120^
CD44v3	Metastasis, EMT	^221^
CD44v4, v5, and v7	Growth and proliferative activity	^108^
CD44v6	EMT, aggressive behavior, poor prognosis,	^34^, ^54^, ^139^, ^223^
CD44v8–v10	Tumorigenic phenotypes, aggressive behavior, poor prognosis, restoration of tumor‐initiating potential, tumor growth	^52^, ^76^, ^83^, ^94^, ^103^, ^119^, ^120^
CD44v9	Progression and metastasis, poor prognosis, EMT	^14^, ^93^, ^101^
CD44v10	Cell proliferation	^199^

### 4.1 MAPK signaling pathway

The activity of the MAPK signaling pathway by CD44 has been associated with the growth of cancer cells.^195^ In CRC patients, miR‐302a binding to CD44 was shown to restore CTX responsiveness by suppressing CSCs‐like properties via EGFR‐mediated MAPK and AKT signaling.^121^ Furthermore, MCF‐7‐A2B1 cells with a high proportion of CD44^+^/CD24^−^/^low^ CSCs were reported to activate the AKT and MAPK pathways via the expression of HNRNPA2B1.^196^ Moreover, although there are few studies on the regulation of CD44 on p38 in cancer, it is well documented that CD44 regulates cancer progression via activation of p38.^197^, ^198^ In a transgenic mouse model of GC, ablation of CD44v was reported to suppress tumor growth via activation of p38 signaling.^120^ In prostate cancer cells, CD44 collaborates with ERBB2 to contribute to the radioresistance of cancer cells via p38 phosphorylation. In breast cancer cells, CD44v10 was found to facilitate cell proliferation via activating ERK/p38 MAPK and AKT/mTOR signaling.^199^ Cell cycle arrest and inhibition of the survival, proliferation, and migration of PC3 cells expressing CD44 and CD133 isolated from prostate cancer by inhibiting p‐p38, p‐ERK, NF‐κB, and PARP.^200^ Furthermore, CD44‐regulated ERK signaling is mostly described as stemness and aggressive behavior of tumors.^201^, ^202^ Yokoyama et al.^132^ found that OSCC cells highly expressing CD44 had been shown to be associated with AMT via activation of ERK. In glioblastoma multiforme cells, high expression of CD44 was found to induce cancer stemness and EMT features via KRAS/ERK signaling activation.^203^ In addition, the high expression of CD44 was capable of inducing ERK phosphorylation, which affects the migratory and invasive potential of lung cancer cells.^204^

### 4.2 Hippo signaling pathway

In the Hippo pathway, CD44 plays key role in cancer stemness and aggressive behavior.^205^, ^206^, ^207^ In lung adenocarcinoma, EDH1 interaction with CD44 was shown to promote CSCs‐like traits, EMT, and metastasis via inhibition of the Hippo pathway.^208^ CD44 upregulation can also confer CSCs‐like properties to malignant mesothelioma cells by activating YAP via Hippo signaling pathway inactivation.^209^ In addition, in docetaxel‐resistant prostate cancer cells, CD44 was reported to promote cell migration and invasion via induction of Hippo–YAP pathways.^210^ Moreover, knockdown YAP partially abolished the stemness of CD44^+^ retinoblastoma stem‐like cells.^211^

### 4.3 Hedgehog signaling pathway

CD44^+^ CSCs is a more appreciated subset which plays key roles in conventional stemness‐related Hedgehog signaling activation.^212^, ^213^ In CD44^+^ HCC, suppressing Hedgehog signaling was reported to reverse sorafenib resistance.^77^ On top of that, in patients with CRC, CD44‐high tumors have been shown to be enriched for the Wnt/β‐catenin and Hedgehog signaling pathways.^214^ Additionally, a bladder CSCs subpopulation with the stem cell‐like marker CD44 was responsive to inhibition of Sonic Hedgehog signaling.^215^ Overall, regulation of the Hedgehog signaling pathway is a promising therapeutic target in CD44^+^ CSCs. CD44^+^ CSCs is associated with activation of Hedgehog signaling.^216^

### 4.4 PI3K‐AKT signaling pathway

A number of studies have also highlighted the effect of CD44 on AKT in relation to cancer cell growth, motility, invasion and stemness.^217^, ^218^, ^219^ Thanee et al.^220^ found that silencing CD44 inhibited cholangiocarcinoma progression and aggressiveness, as well as AKT and mTOR phosphorylation. As demonstrated by GC cell lines and an experimental animal model, chitinase‐like protein CHI3L1 binding to CD44v3 was a specific inducer of activation of ERK, AKT, and β‐catenin signaling and enhancement of GC transfer.^221^ Moreover, studies of in vitro and in vivo found that CD44 promoted HCC migration and extrahepatic metastases mediated by the AKT/ERK signaling CXCR4 axis.^222^ In addition, more recent researches have demonstrated that activation of the PI3K signaling pathway is positively associated with tumor growth and aggressive behavior. In HNSCC, targeting CD44 was found to decrease tumor growth and CSCs by inhibiting PI3K–4EBP1–SOX2 signaling.^52^ Furthermore, in OSCC, silencing CD44v6 diminished invasion/metastasis potential by blocking the PI3K/AKT/GSK3β pathway.^223^ Finally, inhibiting the PI3K–AKT signaling pathway contribute to inhabit invasion and proliferation of glioma cell mediated by epithelial membrane protein 1 (EMP1), which was affected by the expression of CD44.^224^

### 4.5 Twist signaling pathway

Twist is mainly related to EMT in cancer cells,^225^ and CD44 is found to play a key part in the adaptive plasticity of cancer cells by regulating Twist signaling.^226^ CD44 was reported to promote lung CSCs metastasis through Wnt–FoxM1–Twist signaling.^227^ In addition, in colon CSCs with CD133^+^ and CD44^+^ markers, triptolide inhibited cell death, apoptosis and altered cell cycle distribution by inhibiting snail slug and Twist expression, which has been reported to be associated with EMT.^228^ Moreover, in ESCC, the TWIST1–CD44–MMP13 axis has been shown to be involved in tumor aggressiveness and EMT.^229^ On top of that, T mutant allele of CD44 (rs8193C>T) could trigger PKC–Twist, PKC–Nanog, and Nanog–Stat signaling pathways through binding to PUM 2, which is related to prediction of prostate neoplasms and prognosis factor in prostate neoplasms.^230^

### 4.6 HIF signaling pathway

Both HIF‐1α and HIF‐2α play roles in tumor stemness,^231^, ^232^ progression,^233^ and metabolism.^234^ Researches found the expression of CD44 could affect HIF signaling pathway. In glioma, CD44ICD binding to HIF‐2α (but not HIF‐1α) induced stemness via activation of HIF‐targeting genes.^235^ Interestingly, Yang et al.^236^ found HIF‐1α is also associated with MDA‐MB‐231 and 468 cells stemness through Coenzyme Q0 treatment to decrease the expression of CD44. Moreover, in TE10 and TE11 cells, the expression of SET domain‐containing 5 could increase the expressing level of cancer stemness‐related protein HIF‐1α and CD68 expression, which is associated with the coexpression of CD44 and SET domain‐containing 5.^237^ The multiple effects of HIF may associated with the type of cancers, which expressing different interacting factors affected by CD44. In the human gastric cell lines SGC‐7901 and BGC‐823, hypoxia‐increased expression of CD44 was found to increase cell viability and invasion associated with high expression of HIF‐1α.^238^ Additionally, in human breast cancer cells, silencing CD44 was also shown to decrease the glycolytic phenotype of cancer cells via regulating c‐Src/AKT/LKB1/AMPKα/HIF‐1α signaling.^239^

### 4.7 c‐Src signaling pathway

In recent years, although few studies have investigated the c‐Src signaling pathway in cancer regulated by CD44, accumulating evidence suggests that activation of c‐Src plays a key part in tumor progression.^240^, ^241^ In human breast cancer cells, CD44 knockdown suppressed both the mRNA and protein expressing of c‐Src and its downstream MAPK signal.^242^ Moreover, in breast cancer cells, HA‐induced CD44 interacting with c‐Src‐activated Twist resulted in downregulation of tumor suppressor protein, Rho GTPase ROK activation and tumor cell invasion, which are critical prerequisite steps for obtaining metastasis.^243^ Additionally, the TM4SF5/CD44 interaction of metastatic HCC cells activated c‐Src/STAT3/Twist1/Bmi1 signaling, which caused spheroid formation.^244^

### 4.8 Wnt signaling pathway

CD44 is a target gene in the Wnt signaling pathway^245^ and plays a key part in activation of the Wnt pathway mediating chemoresistance,^246^ EMT,^247^ and tumor progression.^248^ In CRC cells, upregulation of CD44, positively associated with the secretory mucin MUC5AC, was reported to confer chemoresistance via β‐catenin/p53/p21.^116^ Moreover, downregulation of RNF128 led to the ubiquitination and degradation of CD44/cortactin, inducing cellular EMT and stemness via activating Wnt/β‐catenin signaling.^124^ Furthermore, in human GC cells MKN45 and KATO III, vitamin D receptor‐induced suppression of CD44 was shown to suppress cell growth, probably via inhibition of Wnt/β‐catenin signaling.^249^

## 5 ANTICANCER THERAPEUTIC STRATEGIES BASED ON TARGETING CD44

### 5.1 Antibodies, peptides, and aptamers

It has been shown that suppressing the expression of CD44^250^ or blocking its interaction with other ligands^251^, ^252^ is a promising strategy in cancer therapy. Antibody against CD44 has been reported to be used in this way in CD44‐positive cancer. For instance, in the human bladder cancer cell line EJ, a novel mouse monoclonal antibody (mAb) KMP1 inhibited proliferation, migration, and adhesion as well as suppressed the xenograft tumor growth in nude mice by blocking CD44.^253^ In OSCC, a defucosylated anti‐CD44 mab 5‐mG2a‐f was found to have antitumor effects both in vitro and in vivo.^254^ Apart from antibodies, another antitumor strategy is to take advantage of synthetic peptides to selectively bind CD44 to inhibit its functions or block the interaction of CD44 and its ligands. In mice harboring tumors, intravenously administered CNLNTIDTC and CNEWQLKSC peptides targeted tumors and inhibited metastasis by binding to CD44v6.^255^ Moreover, it has been demonstrated that a synthetic IGFBP‐3 peptide (215‐KKGFYKKKQCRPSKGRKR‐232) can inhibit the viability of A549 cells as a result of CD44 competing with the HA receptor.^256^ Indeed, a research suggested that CD44 aptamers exert antitumor effects by targeting CD44. In ovarian cancer, an RNA‐based bispecific CD44–EpCAM aptamer was shown to inhibit cell growth and to induce apoptosis by blocking CD44 and EpCAM simultaneously.^257^ Moreover, follow‐up experiments identified the inhibition of orthotopic glioma growth by AS1411 aptamer coloading shikonin and docetaxel targeting CD44‐overexpressing glioma.^258^ Of note, the anticancer effect of antibodies, peptides, and aptamers depends on their binding affinity and specificity to CD44. This suggests a direction for targeted therapy targeting CD44 isoforms and highlights the different roles of the stroma in tumors.

### 5.2 Pharmacological inhibition

Other than directly targeting CD44, several natural compounds and chemotherapeutic agents are also important anticancer agents in cancer cells and CSCs expressing CD44.^259^, ^260^, ^261^ In glioma cells, galangin was found to inhibit EMT and angiogenesis by suppressing the expression of CD44.^262^ Similar results are further supported by Jobani et al.,^263^ who demonstrated that combination treatment with allicin and all‐trans retinoic acid significantly reduced the IC50 value in CD44 expressing melanoma cells CD44. Additionally, in the breast cancer cell line MDA‐MB‐231, ivermectin was shown to preferentially inhibit the CD44^+^/CD24^−^ CSCs subpopulation. Overall, these data suggest that pharmacological therapy that suppresses CD44 expressing in cancer cells is a promising strategy.

### 5.3 Gene therapies

MiRNAs can directly bind to CD44 to silence gene expression at the RNA level, which has been shown to regulate CSCs characteristics and tumor progression.^264^, ^265^, ^266^ For example, in colon cancer cells, miR‐145 and antagomir‐21 were found to suppress CSCs proliferation by inhibiting the expression of CD44.^267^ In addition, another study found that in CRC cells, CTX responsiveness could restored by miR‐302a by inhibiting CD44‐induced CSCs‐like properties.^121^ Moreover, Liu et al.^268^ found miR‐34a could directly target CD44 and miR‐34a inhibited prostate CSCs and metastasis by negatively regulating the expression of CD44. On top of that, Feng et al.^269^ found that miR‐373 and miR‐520s could affect the growth and invasiveness of glioblastoma cells through interacting with CD44 to decrease its expression. In papillary thyroid cancer, miR‐205‐5p/GGCT was found to inhabit its growth and metastasis through regulating the expression of CD44.^270^

On the other hand, it is worth noting that short interfering RNAs (siRNAs) have been shown to knockdown the expression of CD44 via repression of translation to suppress EMT, drug resistance, and growth in tumors.^271^, ^272^, ^273^ In colorectal CSCs, siRNA inhibited EMT‐induced proliferation and invasion by silencing the expression of CD44.^274^ In addition, in the human breast cancer cell line MDA‐MB468, siRNA‐mediated silencing of CD44 was shown to enhance doxorubicin chemosensitivity.^275^ In addition, in the EGFR wild‐type non‐small cell lung cancer cell line H460, knocking down CD44 by siRNA has been found to reduce cell growth and induce cell apoptosis.^276^

Overall, recent developments support the implementation of gene therapy as a new component in therapeutic agents targeting CD44.

### 5.4 Cell therapies

Recently, CD44 isoforms have been found as an promising target for chimeric antigen receptor T cells (CAR T cells) to eliminate CD44 expressing cancer, which is a novel and specific treatment.^277^, ^278^ CD44v6 is found to be associated with tumor progression and aggressive behavior.^279^ Hence, CD44v6 CAR T cells is an attractive therapy to control tumor growth. For instance, CD44v6–CAR T cells were found to specifically lyse CD44v6^+^ acute myeloid leukemia cells associated with cytokine release.^280^ Moreover, Haist et al.^281^ showed a positive link between CD44v6 expression levels and the cytotoxicity of CAR T cells and found that CD44v6–CAR T cells specifically eliminated CD44v6^+^ HNSCC. Furthermore, minicircle DNA‐mediated CD44–CAR T cells were shown to suppress HCC.^282^ Token together, little research has been done on CAR T cells targeting CD44 expressing cancers cells, but it is being rolled out and is a novel therapeutic strategy. CAR T cells targeting CD44s or other CD44v expressing cancer cells require further study, for the reason that highly customized CAR T treatments could focus on the differences of CD44 isoforms expressing in cancer cells to improve their specificity and off‐target effect.

### 5.5 Biological materials

Robust evidence supports that antitumor agents conjugated by biomaterials show good antitumor effects and targeting activity through a ligand–receptor‐mediated targeting mechanism.^283^, ^284^, ^285^ For instance, CS‐conjugated doxorubicin poly(lactic‐co‐glycolic acid) (PLGA) nanoparticles were found to directly target CD44 with low cardiac toxicity and strong antitumor ability.^286^ Moreover, an HA‐labeled PLGA nanoparticle encapsulating both paclitaxel and focal adhesion kinase siRNA was reported to bind to CD44‐positive epithelial ovarian cancer cells to overcome chemoresistance.^287^ In addition, novel HA cross‐linked zein nanogels were developed to deliver curcumin into CT26 cells expressing CD44, which showed high anticancer activity.^288^ In conclusion, biomaterial coupling of antitumor agents shows good target activity and low toxicity, which is a promising therapeutic strategy in cancer treatment. Moreover, a recent research has found a self‐crosslinkable chitosan–HA dialdehyde nanoparticles could target the delivery of siRNA to T24 bladder cancer cells, which could affect bladder cancer through the interaction of CD44 and HA.^289^

## 6 CLINICAL STUDIES

Although accumulating evidence suggests that CD44 plays a key role in the clinicopathological features of numerous cancers,^290^, ^291^ there are relatively few clinical studies designed to evaluate the efficacy of CD44‐targeted therapies in cancer treatment.

To date, 16 clinical studies have been registered on ClinicalTrials. Gov to assess the anticancer effects of targeting CD44. The summary content is shown in Table 3. Among them, three trials are designed to evaluate CD44v6‐specific CAR T‐cell therapies; two trials are using humanized mAb drugs for the treatment of CD44^+^ cancer; two trials are aimed at inhibiting the expression of CD44 in cancer; and one trial is about a drug designed to bind to CD44 for the treatment of chronic lymphocytic leukemia and small lymphocytic lymphoma. However, a phase I trial of an anti‐CD44 humanized antibody, RG7356, in CD44 expressing solid tumor patients was failed.^292^ The study was terminated early because RG7356 does not have a relationship with clinical and/or pharmacodynamic dose–response. On the one hand, in plasma, the RG7536 was converted to a binding‐impaired molecule that cannot result in sufficient antibody levels due to the deamidation of asparaginases in the complementarity determining region of intact antibody. One the other hand, the expression of CD44s or CD44v in patient tumor is crucial. Some CD44s expressing tumor patients (four out of nine) showed tumor shrinkage which was not observed in CD44v expressing tumor patients. This phenomenon may be associated with the iron endocytosis is recompensated by transferrin receptor, resulting in the reducing of drug specific uptake of tumor cells.^30^, ^293^, ^294^

**TABLE 3:** Clinical studies using CD44‐targeting therapies for cancer treatment.

Form of drug	Cancer applications	Interventions	Phase	NCT No.
Gene‐engineered T cells	CD44v6^+^ cancer	CD44v6 CAR T cells	I/II	NCT04427449
	Breast cancer	Her2, GD2, and CD44v6 CAR T cells	I/II	NCT04430595
	CD44v6^+^ acute myeloid leukemia and multiple myeloma	Drug: MLM‐CAR44.1 T cells	I/II	NCT04097301
Humanized mAb	CD44v6^+^ breast neoplasms	Drug: Bivatuzumab mertansine	I	NCT02254005 NCT02254031
	CD44^+^ malignant solid tumors	Drug: RO5429083	I	NCT01358903
CD44v6 inhibitor	Malignant solid tumor	Drug: AMC303	I	NCT03009214
	CD44^+^ ovarian epithelial cancer	Drug: SPL‐108	I	NCT03078400
CD44 binding peptide	Chronic lymphocytic leukemia and small lymphocytic lymphoma	Drug: A6	I	NCT02046928

*Data sources*: ClinicalTrials. Gov.

Together, treatments targeting CD44 are promising, and it is expected that more translational studies will be concentrated on targeting different kinds of CD44 isoforms. Moreover, due to the dominant advantage of combination regimens,^295^, ^296^ more clinic trials about combination regimens should be considered to overcome potential tumor escape or drug resistance.

## 7 CONCLUSIONS

In recent years, there has been an increasing awareness of the complex functions of CD44. A systematic review of landmark CD44‐related studies could facilitate a better understanding of the functional role of CD44 in cancer development and progression and thus will contribute to the development of novel strategies to circumvent their disadvantage and acquire optimal clinical efficacy. Here, we encapsulate the present understanding of the structural and functional roles of CD44 in neoplastic diseases as well as CD44‐regulated signaling pathways. We also discuss current targeting CD44 therapeutic strategies as well as prospective directions for future expansion and highlight existing clinical data supporting its use.

Overall, high expression of CD44, regardless of isoforms, is mainly positively associated with the development of neoplastic disease. Extensive studies have revealed that CD44 mediates cancer initiation and progression through interactions with its ligands, including but not limited to HA, OPN, serglycin, CS, the MMPs family, and FN. As one of the important functions of CD44, its regulation of cancer‐related pathways is pleiotropic, including cancer initiation, aggressive behavior, and stemness. CD44 promotes activation of MAPK, p38, and so on, which is associated with cancer cell stemness, growth, radiation resistance, and cell proliferation. Moreover, CD44 interacting with proteins such as YAP in the Hippo signaling pathway can regulate the stemness and aggressive behavior of cancer. On top of that, as a classical CSCs marker, CD44 regulates cell plasticity through iron mediation.

Expansive evidence indicates that anticancer therapeutic strategies based on targeting CD44 are effective methods, including antibodies, peptides, aptamers, natural and synthetic inhibitors, gene and cell therapy and biomaterials, depending on the interaction of CD44 with its ligands. Among these methods, targeting CD44–CAR T cells is promising. Although it has few applied clinically, its good targeting and durability in CD44 expressing patients show CAR T treatment is worthy of further study. However, clinical trials on CD44 in the treatment of cancer are limited, concentrating on targeting CD44v6, an isoform associated with metastasis and invasiveness. Further studies may focus on other isoforms. On top of that, it is expectable that more translational studies will be conducted to focus on chelating iron or regulate iron concentration to depleting CSCs through regulating the expression of CD44. In addition, clinical evidence suggests that highly expressing CD44 is a potential biomarker, including poor prognosis, tumor grade, and potential malignancy. However, the expression of CD44 was also found to be negatively associated with tumor invasiveness and Gleason grades, which is mainly related to the switch between CD44s and CD44v on the cancer cells through alternative splicing of CD44 isoforms. In conclusion, the conflicting data reports indicate that further study calls for exploring the specific functions of different CD44 isoforms in different kinds of cancers.

## AUTHOR CONTRIBUTIONS

Chenying Fu and Ping Cheng designed this study. Yiming Xu drafted the manuscript. Yiming Xu, Ziyi Bai, and Tianxia Lan revised the manuscript. All authors read and approved the final manuscript.

## CONFLICT OF INTEREST STATEMENT

The authors declare that they have no conflict of interests.

## ETHICS STATEMENT

No ethics approval was required for this review that did not involve patients or patient data.

## Data Availability

Not applicable.

## References

[1] Gomari MM , Farsimadan M , Rostami N , et al. CD44 polymorphisms and its variants, as an inconsistent marker in cancer investigations. Mutat Res Rev Mutat Res. 2021;787:108374. 34083044 10.1016/j.mrrev.2021.108374

[2] Ponta H , Wainwright D , Herrlich P . The CD44 protein family. Int J Biochem Cell Biol. 1998;30(3):299‐305. 9611772 10.1016/s1357-2725(97)00152-0

[3] Liu S , Liu Z , Shang A , et al. CD44 is a potential immunotherapeutic target and affects macrophage infiltration leading to poor prognosis. Sci Rep. 2023;13(1):9657. 37316699 10.1038/s41598-023-33915-4 PMC10267145

[4] Gupta RC , Lall R , Srivastava A , Sinha A . Hyaluronic acid: molecular mechanisms and therapeutic trajectory. Front Vet Sci. 2019;6:192. 31294035 10.3389/fvets.2019.00192 PMC6603175

[5] Chen S , Zhang M , Li J , et al. β‐catenin‐controlled tubular cell‐derived exosomes play a key role in fibroblast activation via the OPN‐CD44 axis. J Extracell Vesicles. 2022;11(3):e12203. 35312232 10.1002/jev2.12203 PMC8936047

[6] Chu Q , Huang H , Huang T , et al. Extracellular serglycin upregulates the CD44 receptor in an autocrine manner to maintain self‐renewal in nasopharyngeal carcinoma cells by reciprocally activating the MAPK/β‐catenin axis. Cell Death Dis. 2016;7(11):e2456. 27809309 10.1038/cddis.2016.287 PMC5260886

[7] Amorim S , Reis CA , Reis RL , Pires RA . Extracellular matrix mimics using hyaluronan‐based biomaterials. Trends Biotechnol. 2021;39(1):90‐104. 32654775 10.1016/j.tibtech.2020.06.003

[8] Chaudhry G‐ES , Akim A , Naveed Zafar M , Safdar N , Sung YY , Muhammad TST . Understanding hyaluronan receptor (CD44) interaction, HA‐CD44 activated potential targets in cancer therapeutics. Adv Pharm Bull. 2021;11(3):426‐438. 34513617 10.34172/apb.2021.050 PMC8421618

[9] Chen C , Zhao S , Karnad A , Freeman JW . The biology and role of CD44 in cancer progression: therapeutic implications. J Hematol Oncol. 2018;11(1):1‐23. 29747682 10.1186/s13045-018-0605-5 PMC5946470

[10] Yang C , Sheng Y , Shi X , et al. CD44/HA signaling mediates acquired resistance to a PI3Kα inhibitor. Cell Death Dis. 2020;11(10):831. 33024087 10.1038/s41419-020-03037-0 PMC7538592

[11] Farahani DB , Akrami H , Moradi B , Mehdizadeh K , Fattahi MR . The effect of hsa‐miR‐451b knockdown on biological functions of gastric cancer stem‐like cells. Biochem Genet. 2021;59(5):1203‐1224. 33725258 10.1007/s10528-021-10057-8

[12] Li L , Hao X , Qin J , et al. Antibody against CD44s inhibits pancreatic tumor initiation and postradiation recurrence in mice. Gastroenterology. 2014;146(4):1108‐1118. 24397969 10.1053/j.gastro.2013.12.035 PMC3982149

[13] Brown RL , Reinke LM , Damerow MS , et al. CD44 splice isoform switching in human and mouse epithelium is essential for epithelial‐mesenchymal transition and breast cancer progression. J Clin Invest. 2011;121(3):1064‐1074. 21393860 10.1172/JCI44540 PMC3049398

[14] Todaro M , Gaggianesi M , Catalano V , et al. CD44v6 is a marker of constitutive and reprogrammed cancer stem cells driving colon cancer metastasis. Cell Stem Cell. 2014;14(3):342‐356. 24607406 10.1016/j.stem.2014.01.009

[15] Wang SJ , Wreesmann VB , Bourguignon LYW . Association of CD44 V3‐containing isoforms with tumor cell growth, migration, matrix metalloproteinase expression, and lymph node metastasis in head and neck cancer. Head Neck. 2007;29(6):550‐558. 17252589 10.1002/hed.20544

[16] Kolliopoulos C , Lin C‐Y , Heldin C‐H , Moustakas A , Heldin P . Has2 natural antisense RNA and Hmga2 promote Has2 expression during TGFβ‐induced EMT in breast cancer. Matrix Biol. 2019;80:29‐45. 30194979 10.1016/j.matbio.2018.09.002

[17] Huang C , Yoon C , Zhou X‐H , et al. ERK1/2‐Nanog signaling pathway enhances CD44(+) cancer stem‐like cell phenotypes and epithelial‐to‐mesenchymal transition in head and neck squamous cell carcinomas. Cell Death Dis. 2020;11(4):266. 32327629 10.1038/s41419-020-2448-6 PMC7181750

[18] Ji H , Kong L , Wang Y , et al. CD44 expression is correlated with osteosarcoma cell progression and immune infiltration and affects the Wnt/β‐catenin signaling pathway. J Bone Oncol. 2023;41:100487. 37287706 10.1016/j.jbo.2023.100487 PMC10242553

[19] Ahmad SMS , Nazar H , Rahman MM , Rusyniak RS , Ouhtit A . ITGB1BP1, a novel transcriptional target of cd44‐downstream signaling promoting cancer cell invasion. Breast Cancer (Dove Med Press). 2023;15:373‐380. 37252376 10.2147/BCTT.S404565 PMC10225144

[20] Li D , Wang X , Han K , et al. Hypoxia and CD44 receptors dual‐targeted nano‐micelles with AGT‐inhibitory activity for the targeting delivery of carmustine. Int J Biol Macromol. 2023;246:125657. 37399878 10.1016/j.ijbiomac.2023.125657

[21] Chen K‐L , Li D , Lu T‐X , Chang S‐W . Structural characterization of the CD44 stem region for standard and cancer‐associated isoforms. Int J Mol Sci. 2020;21(1):336. 31947887 10.3390/ijms21010336 PMC6982006

[22] Karousou E , Misra S , Ghatak S , et al. Roles and targeting of the HAS/hyaluronan/CD44 molecular system in cancer. Matrix Biol. 2017;59:3‐22. 27746219 10.1016/j.matbio.2016.10.001

[23] Hassn Mesrati M , Syafruddin SE , Mohtar MA , Syahir A . CD44: a multifunctional mediator of cancer progression. Biomolecules. 2021;11(12):1850. 34944493 10.3390/biom11121850 PMC8699317

[24] Tölg C , Hofmann M , Herrlich P , Ponta H . Splicing choice from ten variant exons establishes CD44 variability. Nucleic Acids Res. 1993;21(5):1225‐1229. 8464707 10.1093/nar/21.5.1225 PMC309286

[25] Mishra MN , Chandavarkar V , Sharma R , Bhargava D . Structure, function and role of CD44 in neoplasia. J Oral Maxillofac Pathol. 2019;23(2):267‐272. 31516234 10.4103/jomfp.JOMFP_246_18 PMC6714250

[26] Sneath RJ , Mangham DC . The normal structure and function of CD44 and its role in neoplasia. Mol Pathol. 1998;51(4):191‐200. 9893744 10.1136/mp.51.4.191 PMC395635

[27] Muys BR , Anastasakis DG , Claypool D , et al. The p53‐induced RNA‐binding protein ZMAT3 is a splicing regulator that inhibits the splicing of oncogenic CD44 variants in colorectal carcinoma. Genes Dev. 2021;35(1‐2):102‐116. 33334821 10.1101/gad.342634.120 PMC7778265

[28] Bennett KL , Jackson DG , Simon JC , et al. CD44 isoforms containing exon V3 are responsible for the presentation of heparin‐binding growth factor. J Cell Biol. 1995;128(4):687‐698. 7532176 10.1083/jcb.128.4.687 PMC2199889

[29] Basuli D , Tesfay L , Deng Z , et al. Iron addiction: a novel therapeutic target in ovarian cancer. Oncogene. 2017;36(29):4089‐4099. 28319068 10.1038/onc.2017.11 PMC5540148

[30] Müller S , Sindikubwabo F , Cañeque T , et al. CD44 regulates epigenetic plasticity by mediating iron endocytosis. Nat Chem. 2020;12(10):929‐938. 32747755 10.1038/s41557-020-0513-5 PMC7612580

[31] Guo Q , Yang C , Gao F . The state of CD44 activation in cancer progression and therapeutic targeting. FEBS J. 2022;289(24):7970‐7986. 34478583 10.1111/febs.16179

[32] Piselli P , Vendetti S , Vismara D , et al. Different expression of CD44, ICAM‐1, and HSP60 on primary tumor and metastases of a human pancreatic carcinoma growing in scid mice. Anticancer Res. 2000;20(2A):825‐831. 10810361

[33] Zhang F‐L , Cao J‐L , Xie H‐Y , et al. Cancer‐associated MORC2‐mutant M276I regulates an hnRNPM‐mediated CD44 splicing switch to promote invasion and metastasis in triple‐negative breast cancer. Cancer Res. 2018;78(20):5780‐5792. 30093560 10.1158/0008-5472.CAN-17-1394

[34] da Cunha CB , Klumpers DD , Koshy ST , et al. CD44 alternative splicing in gastric cancer cells is regulated by culture dimensionality and matrix stiffness. Biomaterials. 2016;98:152‐162. 27187279 10.1016/j.biomaterials.2016.04.016

[35] Espejo‐Román JM , Rubio‐Ruiz B , Chayah‐Ghaddab M , et al. N‐aryltetrahydroisoquinoline derivatives as HA‐CD44 interaction inhibitors: design, synthesis, computational studies, and antitumor effect. Eur J Med Chem. 2023;258:115570. 37413883 10.1016/j.ejmech.2023.115570

[36] Chen L , Huan X , Xiao G‐H , et al. Osteopontin and its downstream carcinogenic molecules: regulatory mechanisms and prognostic value in cancer progression. Neoplasma. 2022;69(6):1253‐1269. 35951454 10.4149/neo_2022_220507N489

[37] Liang S , Duan Y , Xing Z , et al. Inhibition of cell proliferation and migration by chondroitin sulfate‐g‐polyethylenimine‐mediated miR‐34a delivery. Colloids Surf B Biointerfaces. 2015;136:577‐584. 26454548 10.1016/j.colsurfb.2015.09.054

[38] Morath I , Hartmann TN , Orian‐Rousseau V . CD44: More than a mere stem cell marker. Int J Biochem Cell Biol. 2016;81(Pt A):166‐173. 27640754 10.1016/j.biocel.2016.09.009

[39] Wu G , Song X , Liu J , et al. Expression of CD44 and the survival in glioma: a meta‐analysis. Biosci Rep. 2020;40(4):BSR20200520. 32232385 10.1042/BSR20200520 PMC7160241

[40] Kolliopoulos C , Ali MM , Castillejo‐Lopez C , Heldin C‐H , Heldin P . CD44 depletion in glioblastoma cells suppresses growth and stemness and induces senescence. Cancers (Basel). 2022;14(15):3747. 35954411 10.3390/cancers14153747 PMC9367353

[41] Zhang H , Cao H , Luo H , et al. RUNX1/CD44 axis regulates the proliferation, migration, and immunotherapy of gliomas: a single‐cell sequencing analysis. Front Immunol. 2023;14:1086280. 36776876 10.3389/fimmu.2023.1086280 PMC9909339

[42] Kamamoto D , Saga I , Ohara K , Yoshida K , Sasaki H . Association between CD133, CD44, and nestin expression and prognostic factors in high‐grade meningioma. World Neurosurg. 2018; S1878‐8750 (18) 32890‐0. 10.1016/j.wneu.2018.12.067 30593958

[43] Li HZ , Gong HD , Wang C , Li JK . The role of osteopontin and its receptor in meningioma development and progression. J Biol Regul Homeost Agents. 2018;32(1):69‐74. 29504367

[44] Wang C , Wang Z , Chen C , et al. A low MW inhibitor of CD44 dimerization for the treatment of glioblastoma. Br J Pharmacol. 2020;177(13):3009‐3023. 32080830 10.1111/bph.15030 PMC7280016

[45] Loreth D , Schuette M , Zinke J , et al. CD74 and CD44 expression on CTCs in cancer patients with brain metastasis. Int J Mol Sci. 2021;22(13):6993. 34209696 10.3390/ijms22136993 PMC8268634

[46] Huang Q , Liu L , Xiao D , et al. CD44+ lung cancer stem cell‐derived pericyte‐like cells cause brain metastases through GPR124‐enhanced trans‐endothelial migration. Cancer Cell. 2023;41(9):1621‐1636.e8. 37595587 10.1016/j.ccell.2023.07.012

[47] Martins Gama J , Caetano Oliveira R , Teixeira P , et al. An immunohistochemical study of breast cancer brain metastases: the role of CD44 and AKT in the prognosis. Appl Immunohistochem Mol Morphol. 2023;31(5):318‐323. 37093706 10.1097/PAI.0000000000001119

[48] Heft Neal ME , Brenner JC , Prince MEP , Chinn SB . Advancement in cancer stem cell biology and precision medicine‐review article head and neck cancer stem cell plasticity and the tumor microenvironment. Front Cell Dev Biol. 2021;9:660210. 35047489 10.3389/fcell.2021.660210 PMC8762309

[49] Leinung M , Ernst B , Döring C , et al. Expression of ALDH1A1 and CD44 in primary head and neck squamous cell carcinoma and their value for carcinogenesis, tumor progression and cancer stem cell identification. Oncol Lett. 2015;10(4):2289‐2294. 26622836 10.3892/ol.2015.3542 PMC4579825

[50] Gomez KE , Wu F , Keysar SB , et al. Cancer Cell CD44 Mediates Macrophage/Monocyte‐Driven Regulation of Head and Neck Cancer Stem CellsCD44 and Macrophages Regulate HNSCC Stem Cells. Cancer research. 2020;80(19):4185‐4198. 32816856 10.1158/0008-5472.CAN-20-1079 PMC8146866

[51] Liu H , Rokana T , Kawaguchi M , et al. Homophilic CD44 interactions mediate tumor cell aggregation and polyclonal metastasis in patient‐derived breast cancer models. Cancer Discov. 2019;9(1):96‐113. 30361447 10.1158/2159-8290.CD-18-0065 PMC6328322

[52] Gomez KE , Wu F , Keysar SB , et al. Cancer cell CD44 mediates macrophage/monocyte‐driven regulation of head and neck cancer stem cells CD44 and macrophages regulate HNSCC stem cells. Cancer Res. 2020;80(19):4185‐4198. 32816856 10.1158/0008-5472.CAN-20-1079 PMC8146866

[53] Ludwig N , Szczepanski MJ , Gluszko A , et al. CD44 (+) tumor cells promote early angiogenesis in head and neck squamous cell carcinoma. Cancer Lett. 2019;467:85‐95. 31593802 10.1016/j.canlet.2019.10.010

[54] Odenthal J , Rijpkema M , Bos D , et al. Targeting CD44v6 for fluorescence‐guided surgery in head and neck squamous cell carcinoma. Sci Rep. 2018;8(1):10467. 29992954 10.1038/s41598-018-28059-9 PMC6041314

[55] Choi J‐H , Lee B‐S , Jang JY , et al. Single‐cell transcriptome profiling of the stepwise progression of head and neck cancer. Nat Commun. 2023;14(1):1055. 36828832 10.1038/s41467-023-36691-x PMC9958029

[56] Escudero Mendez L , Srinivasan M , Hamouda RK , et al. Evaluation of CD44+/CD24‐ and aldehyde dehydrogenase enzyme markers in cancer stem cells as prognostic indicators for triple‐negative breast cancer. Cureus. 2022;14(8):e28056. 36120232 10.7759/cureus.28056 PMC9476834

[57] Alateyah N , Gupta I , Rusyniak RS , Ouhtit A . SOD2, a potential transcriptional target underpinning CD44‐promoted breast cancer progression. molecules. 2022;27(3):811. 35164076 10.3390/molecules27030811 PMC8839817

[58] Stevens LE , Peluffo G , Qiu X , et al. JAK‐STAT signaling in inflammatory breast cancer enables chemotherapy‐resistant cell states. Cancer Res. 2023;83(2):264‐284. 36409824 10.1158/0008-5472.CAN-22-0423 PMC9845989

[59] Dashzeveg NK , Jia Y , Zhang Y , et al. Dynamic glycoprotein hyposialylation promotes chemotherapy evasion and metastatic seeding of quiescent circulating tumor cell clusters in breast cancer. Cancer Discov. 2023;13(9):2050‐2071. 37272843 10.1158/2159-8290.CD-22-0644 PMC10481132

[60] Gu J , Chen D , Li Z , Yang Y , Ma Z , Huang G . Prognosis assessment of CD44+/CD24‐ in breast cancer patients: a systematic review and meta‐analysis. Arch Gynecol Obstet. 2022;306(4):1147‐1160. 35435483 10.1007/s00404-022-06402-w

[61] Gao R , Li D , Xun J , et al. CD44ICD promotes breast cancer stemness via PFKFB4‐mediated glucose metabolism. Theranostics. 2018;8(22):6248. 30613295 10.7150/thno.28721 PMC6299690

[62] Zhang H , Brown RL , Wei Y , et al. CD44 splice isoform switching determines breast cancer stem cell state. Genes Dev. 2019;33(3‐4):166‐179. 30692202 10.1101/gad.319889.118 PMC6362815

[63] Yang C , Cao M , Liu Y , et al. Inducible formation of leader cells driven by CD44 switching gives rise to collective invasion and metastases in luminal breast carcinomas. Oncogene. 2019;38(46):7113‐7132. 31417182 10.1038/s41388-019-0899-y

[64] Bei Y , Cheng N , Chen T , et al. CDK5 inhibition abrogates TNBC stem‐cell property and enhances anti‐PD‐1 therapy. Adv Sci. 2020;7(22):2001417. 10.1002/advs.202001417 PMC7675186 33240752

[65] Ma J , Wu R , Chen Z , et al. CD44 is a prognostic biomarker correlated with immune infiltrates and metastasis in clear cell renal cell carcinoma. Anticancer Res. 2023;43(8):3493‐3506. 37500138 10.21873/anticanres.16526

[66] Ma J , Li M , Chai J , et al. Expression of RSK4, CD44 and MMP‐9 is upregulated and positively correlated in metastatic ccRCC. Diagn Pathol. 2020;15(1):1‐10. 32209138 10.1186/s13000-020-00948-6 PMC7093975

[67] Sekino Y , Han X , Kobayashi G , et al. BUB1B overexpression is an independent prognostic marker and associated with CD44, p53, and PD‐L1 in renal cell carcinoma. Oncology. 2021;99(4):240‐250. 33588420 10.1159/000512446

[68] Kozawa K , Sekai M , Ohba K , et al. The CD44/COL17A1 pathway promotes the formation of multilayered, transformed epithelia. Curr Biol. 2021;31(14):3086‐3097.e7. 34087104 10.1016/j.cub.2021.04.078

[69] Li D , Liu S , Xu J , et al. Ferroptosis‐related gene CHAC1 is a valid indicator for the poor prognosis of kidney renal clear cell carcinoma. J Cell Mol Med. 2021;25(7):3610‐3621. 33728749 10.1111/jcmm.16458 PMC8034464

[70] Xu F , Guan Y , Xue L , et al. The effect of a novel glycolysis‐related gene signature on progression, prognosis and immune microenvironment of renal cell carcinoma. BMC Cancer. 2020;20(1):1207. 33287763 10.1186/s12885-020-07702-7 PMC7720455

[71] Fiedorowicz M , Khan MI , Strzemecki D , et al. Renal carcinoma CD105‐/CD44‐ cells display stem‐like properties in vitro and form aggressive tumors in vivo. Sci Rep. 2020;10(1):5379. 32214151 10.1038/s41598-020-62205-6 PMC7096525

[72] Liu Y‐C , Yeh C‐T , Lin K‐H . Cancer stem cell functions in hepatocellular carcinoma and comprehensive therapeutic strategies. Cells. 2020;9(6):1331. 32466488 10.3390/cells9061331 PMC7349579

[73] Zarębska I , Gzil A , Durślewicz J , et al. The clinical, prognostic and therapeutic significance of liver cancer stem cells and their markers. Clin Res Hepatol Gastroenterol. 2021;45(3):101664. 33667731 10.1016/j.clinre.2021.101664

[74] Asai R , Tsuchiya H , Amisaki M , et al. CD44 standard isoform is involved in maintenance of cancer stem cells of a hepatocellular carcinoma cell line. Cancer Med. 2019;8(2):773‐782. 30636370 10.1002/cam4.1968 PMC6382709

[75] Dhar D , Antonucci L , Nakagawa H , et al. Liver cancer initiation requires p53 inhibition by CD44‐enhanced growth factor signaling. Cancer Cell. 2018;33(6):1061‐1077.e6. 29894692 10.1016/j.ccell.2018.05.003 PMC6005359

[76] Jun SY , Yoon HR , Yoon J‐Y , et al. The human TOR signaling regulator is the key indicator of liver cancer patients' overall survival: TIPRL/LC3/CD133/CD44 as potential biomarkers for early liver cancers. Cancers (Basel). 2021;13(12):2925. 34208132 10.3390/cancers13122925 PMC8230774

[77] Wang S , Wang Y , Xun X , et al. Hedgehog signaling promotes sorafenib resistance in hepatocellular carcinoma patient‐derived organoids. J Exp Clin Cancer Res. 2020;39(1):22. 31992334 10.1186/s13046-020-1523-2 PMC6986013

[78] Ho DW‐H , Tsui Y‐M , Sze KM‐F , et al. Single‐cell transcriptomics reveals the landscape of intra‐tumoral heterogeneity and stemness‐related subpopulations in liver cancer. Cancer Lett. 2019;459:176‐185. 31195060 10.1016/j.canlet.2019.06.002

[79] Toh TB , Lim JJ , Hooi L , Rashid MBMA , Chow EK‐H . Targeting Jak/Stat pathway as a therapeutic strategy against SP/CD44+ tumorigenic cells in Akt/β‐catenin‐driven hepatocellular carcinoma. J Hepatol. 2020;72(1):104‐118. 31541681 10.1016/j.jhep.2019.08.035

[80] Bishnupuri KS , Sainathan SK , Ciorba MA , Houchen CW , Dieckgraefe BK . Reg4 interacts with CD44 to regulate proliferation and stemness of colorectal and pancreatic cancer cells. Mol Cancer Res. 2022;20(3):387‐399. 34753802 10.1158/1541-7786.MCR-21-0224

[81] Gzil A , Zarębska I , Bursiewicz W , Antosik P , Grzanka D , Szylberg Ł. Markers of pancreatic cancer stem cells and their clinical and therapeutic implications. Mol Biol Rep. 2019;46(6):6629‐6645. 31486978 10.1007/s11033-019-05058-1

[82] Leon F , Seshacharyulu P , Nimmakayala RK , et al. Reduction in O‐glycome induces differentially glycosylated CD44 to promote stemness and metastasis in pancreatic cancer. Oncogene. 2022;41(1):57‐71. 34675409 10.1038/s41388-021-02047-2 PMC8727507

[83] Xie Z , Gao Y , Ho C , et al. Exosome‐delivered CD44v6/C1QBP complex drives pancreatic cancer liver metastasis by promoting fibrotic liver microenvironment. Gut. 2022;71(3):568‐579. 33827783 10.1136/gutjnl-2020-323014

[84] Kumar S , Inigo JR , Kumar R , et al. Nimbolide reduces CD44 positive cell population and induces mitochondrial apoptosis in pancreatic cancer cells. Cancer Lett. 2018;413:82‐93. 29107110 10.1016/j.canlet.2017.10.029 PMC5706561

[85] Liu Y , Wu T , Lu D , Zhen J , Zhang L . CD44 overexpression related to lymph node metastasis and poor prognosis of pancreatic cancer. Int J Biol Markers. 2018;33(3):308‐313. 29683068 10.1177/1724600817746951

[86] Wang D , Zhu H , Zhu Y , et al. Retraction notice to “CD133+/CD44+/Oct4+/Nestin+ stem‐like cells isolated from Panc‐1 cell line may contribute to multi‐resistance and metastasis of pancreatic cancer” [Acta Histochemica 115 (2013) 349‐356]. Acta Histochem. 2018;120(3):302. 29598902 10.1016/j.acthis.2018.03.005

[87] Spadea A , de la Rosa JMR , Tirella A , et al. Evaluating the efficiency of hyaluronic acid for tumor targeting via CD44. Mol Pharm. 2019;16(6):2481‐2493. 31013093 10.1021/acs.molpharmaceut.9b00083

[88] Kesharwani P , Banerjee S , Padhye S , Sarkar FH , Iyer AK . Hyaluronic acid engineered nanomicelles loaded with 3,4‐difluorobenzylidene curcumin for targeted killing of CD44+ stem‐like pancreatic cancer cells. Biomacromolecules. 2015;16(9):3042‐3053. 26302089 10.1021/acs.biomac.5b00941

[89] Kesharwani P , Xie L , Banerjee S , et al. Hyaluronic acid‐conjugated polyamidoamine dendrimers for targeted delivery of 3, 4‐difluorobenzylidene curcumin to CD44 overexpressing pancreatic cancer cells. Colloids Surf B. 2015;136:413‐423. 10.1016/j.colsurfb.2015.09.043 26440757

[90] Kang X , Bu F , Feng W , et al. Dual‐cascade responsive nanoparticles enhance pancreatic cancer therapy by eliminating tumor‐resident intracellular bacteria. Adv Mater. 2022;34(49):e2206765. 36082582 10.1002/adma.202206765

[91] Qian L , Su H , Wang G , Li B , Shen G , Gao Q . Anti‐tumor activity of bufalin by inhibiting c‐MET mediated MEK/ERK and PI3K/AKT signaling pathways in gallbladder cancer. J Cancer. 2020;11(11):3114‐3123. 32231716 10.7150/jca.38393 PMC7097950

[92] Kalekou H , Miliaras D . Immunohistochemical study of microvessel density, CD44 (standard form), p53 protein and c‐erbB2 in gallbladder carcinoma. J Gastroenterol Hepatol. 2004;19(7):812‐818. 15209630 10.1111/j.1440-1746.2004.03357.x

[93] Miwa T , Nagata T , Kojima H , Sekine S , Okumura T . Isoform switch of CD44 induces different chemotactic and tumorigenic ability in gallbladder cancer. Int J Oncol. 2017;51(3):771‐780. 28677740 10.3892/ijo.2017.4063 PMC5564409

[94] Yamaguchi A , Zhang M , Goi T , et al. Expression of variant CD44 containing variant exon v8‐10 in gallbladder cancer. Oncol Rep. 2000;7(3):541‐544. 10767365 10.3892/or.7.3.541

[95] Yin B‐B , Wu S‐J , Zong H‐J , Ma B‐J , Cai D . Preliminary screening and identification of stem cell‐like sphere clones in a gallbladder cancer cell line GBC‐SD. J Zhejiang Univ Sci B. 2011;12(4):256‐263. 21462380 10.1631/jzus.B1000303 PMC3072470

[96] Lai J , Yang S , Lin Z , et al. Update on chemoresistance mechanisms to first‐line chemotherapy for gallbladder cancer and potential reversal strategies. Am J Clin Oncol. 2023;46(4):131‐141. 36867653 10.1097/COC.0000000000000989 PMC10030176

[97] Khales SA , Mozaffari‐Jovin S , Geerts D , Abbaszadegan MR . TWIST1 activates cancer stem cell marker genes to promote epithelial‐mesenchymal transition and tumorigenesis in esophageal squamous cell carcinoma. BMC Cancer. 2022;22(1):1272. 36474162 10.1186/s12885-022-10252-9 PMC9724315

[98] Tsuchihashi K , Hirata Y , Yamasaki J , et al. Presence of spontaneous epithelial‐mesenchymal plasticity in esophageal cancer. Biochem Biophys Rep. 2022;30:101246. 35330672 10.1016/j.bbrep.2022.101246 PMC8938278

[99] Horitani S , Fukui T , Tanimura Y , et al. Specific Smad2/3 linker phosphorylation indicates esophageal non‐neoplastic and neoplastic stem‐like cells and neoplastic development. Dig Dis Sci. 2021;66(6):1862‐1874. 32705438 10.1007/s10620-020-06489-8

[100] Moghbeli M , Mosannen Mozaffari H , Memar B , Forghanifard MM , Gholamin M , Abbaszadegan MR . Role of MAML1 in targeted therapy against the esophageal cancer stem cells. J Transl Med. 2019;17(1):1‐12. 30992079 10.1186/s12967-019-1876-5 PMC6469193

[101] Taniguchi D , Saeki H , Nakashima Y , et al. CD44v9 is associated with epithelial‐mesenchymal transition and poor outcomes in esophageal squamous cell carcinoma. Cancer Med. 2018;7(12):6258‐6268. 30474922 10.1002/cam4.1874 PMC6308082

[102] Zuo J , Zhu K , Wang Y , Yu Z . MicroRNA‐34a suppresses invasion and metastatic in esophageal squamous cell carcinoma by regulating CD44. Mol Cell Biochem. 2018;443(1‐2):139‐149. 29094237 10.1007/s11010-017-3218-3

[103] Yu X‐M , Li S‐J , Yao Z‐T , et al. N4‐acetylcytidine modification of lncRNA CTC‐490G23.2 promotes cancer metastasis through interacting with PTBP1 to increase CD44 alternative splicing. Oncogene. 2023;42(14):1101‐1116. 36792757 10.1038/s41388-023-02628-3

[104] Li K , Sun X , Zha R , et al. Counterintuitive production of tumor‐suppressive secretomes from Oct4‐ and c‐Myc‐overexpressing tumor cells and MSCs. Theranostics. 2022;12(7):3084‐3103. 35547745 10.7150/thno.70549 PMC9065181

[105] Wang X , Cai J , Zhao L , et al. NUMB suppression by miR‐9‐5P enhances CD44+ prostate cancer stem cell growth and metastasis. Sci Rep. 2021;11(1):11210. 34045601 10.1038/s41598-021-90700-x PMC8160147

[106] Koukourakis IM , Platoni K , Kouloulias V , Arelaki S , Zygogianni A . Prostate cancer stem cells: biology and treatment implications. Int J Mol Sci. 2023;24(19):14890. 37834336 10.3390/ijms241914890 PMC10573523

[107] Chen Q , Gu M , Cai Z‐K , et al. TGF‐β1 promotes epithelial‐to‐mesenchymal transition and stemness of prostate cancer cells by inducing PCBP1 degradation and alternative splicing of CD44. Cell Mol Life Sci. 2021;78(3):949‐962. 32440711 10.1007/s00018-020-03544-5 PMC11072728

[108] Rutz J , Thaler S , Maxeiner S , Chun FKH , Blaheta RA . Sulforaphane reduces prostate cancer cell growth and proliferation in vitro by modulating the Cdk‐cyclin axis and expression of the CD44 variants 4, 5, and 7. Int J Mol Sci. 2020;21(22):8724. 33218199 10.3390/ijms21228724 PMC7699211

[109] Wróbel T , Luty M , Catapano J , et al. CD44+ cells determine fenofibrate‐induced microevolution of drug‐resistance in prostate cancer cell populations. Stem Cells. 2020;38(12):1544‐1556. 32985018 10.1002/stem.3281 PMC7756969

[110] Liu Z , Wang L , Xu H , et al. Heterogeneous responses to mechanical force of prostate cancer cells inducing different metastasis patterns. Adv Sci. 2020;7(15):1903583. 10.1002/advs.201903583 PMC7404165 32775149

[111] Chinnapaka S , Bakthavachalam V , Munirathinam G . Repurposing antidepressant sertraline as a pharmacological drug to target prostate cancer stem cells: dual activation of apoptosis and autophagy signaling by deregulating redox balance. Am J Cancer Res. 2020;10(7):2043‐2065. 32775000 PMC7407340

[112] Verma S , Shankar E , Kalayci FNC , et al. Androgen deprivation induces transcriptional reprogramming in prostate cancer cells to develop stem cell‐like characteristics. Int J Mol Sci. 2020;21(24):9568. 33339129 10.3390/ijms21249568 PMC7765584

[113] Li J , Pu T , Yin L , Li Q , Liao C‐P , Wu BJ . MAOA‐mediated reprogramming of stromal fibroblasts promotes prostate tumorigenesis and cancer stemness. Oncogene. 2020;39(16):3305‐3321. 32066880 10.1038/s41388-020-1217-4

[114] Hou W , Kong L , Hou Z , Ji H . CD44 is a prognostic biomarker and correlated with immune infiltrates in gastric cancer. BMC Med Genomics. 2022;15(1):225. 36316684 10.1186/s12920-022-01383-w PMC9620622

[115] Fujiwara‐Tani R , Sasaki T , Ohmori H , et al. Concurrent expression of CD47 and CD44 in colorectal cancer promotes malignancy. Pathobiology. 2019;86(4):182‐189. 31132784 10.1159/000496027

[116] Pothuraju R , Rachagani S , Krishn SR , et al. Molecular implications of MUC5AC‐CD44 axis in colorectal cancer progression and chemoresistance. Mol Cancer. 2020;19(1):37. 32098629 10.1186/s12943-020-01156-y PMC7041280

[117] Wei F , Zhang T , Deng S‐C , et al. PD‐L1 promotes colorectal cancer stem cell expansion by activating HMGA1‐dependent signaling pathways. Cancer Lett. 2019;450:1‐13. 30776481 10.1016/j.canlet.2019.02.022

[118] Wu C , Ding H , Wang S , et al. DAXX inhibits cancer stemness and epithelial–mesenchymal transition in gastric cancer. Br J Cancer. 2020;122(10):1477‐1485. 32203224 10.1038/s41416-020-0800-3 PMC7217831

[119] Lau WM , Teng E , Chong HS , et al. CD44v8‐10 is a cancer‐specific marker for gastric cancer stem cells. Cancer Res. 2014;74(9):2630‐2641. 24618343 10.1158/0008-5472.CAN-13-2309

[120] Ishimoto T , Nagano O , Yae T , et al. CD44 variant regulates redox status in cancer cells by stabilizing the xCT subunit of system xc(‐) and thereby promotes tumor growth. Cancer Cell. 2011;19(3):387‐400. 21397861 10.1016/j.ccr.2011.01.038

[121] Sun L , Fang Y , Wang X , et al. miR‐302a inhibits metastasis and cetuximab resistance in colorectal cancer by targeting NFIB and CD44. Theranostics. 2019;9(26):8409. 31754405 10.7150/thno.36605 PMC6857048

[122] Weitzenböck HP , Gschwendtner A , Wiesner C , et al. Proteome analysis of NRF2 inhibition in melanoma reveals CD44 up‐regulation and increased apoptosis resistance upon vemurafenib treatment. Cancer Med. 2022;11(4):956‐967. 34951143 10.1002/cam4.4506 PMC8855890

[123] Mohammadi A , Najafi S , Amini M , Baradaran B , Firouzamandi M . B7H6 silencing increases chemosensitivity to dacarbazine and suppresses cell survival and migration in cutaneous melanoma. Melanoma Res. 2023;33(3):173‐183. 37053079 10.1097/CMR.0000000000000890

[124] Wei C‐Y , Zhu M‐X , Yang Y‐W , et al. Downregulation of RNF128 activates Wnt/β‐catenin signaling to induce cellular EMT and stemness via CD44 and CTTN ubiquitination in melanoma. J Hematol Oncol. 2019;12(1):21. 30832692 10.1186/s13045-019-0711-z PMC6399928

[125] Sadeghi RS , Kulej K , Kathayat RS , et al. Wnt5a signaling induced phosphorylation increases APT1 activity and promotes melanoma metastatic behavior. eLife. 2018;7:e34362. 29648538 10.7554/eLife.34362 PMC5919757

[126] Wu R‐L , Sedlmeier G , Kyjacova L , et al. Hyaluronic acid‐CD44 interactions promote BMP4/7‐dependent Id1/3 expression in melanoma cells. Sci Rep. 2018;8(1):14913. 30297743 10.1038/s41598-018-33337-7 PMC6175841

[127] Li Y , Hou H , Liu Z , et al. CD44 targeting nanodrug based on chondroitin sulfate for melanoma therapy by inducing mitochondrial apoptosis pathways. Carbohydr Polym. 2023;320:121255. 37659829 10.1016/j.carbpol.2023.121255

[128] Hu W , Zhao Y , Su L , et al. Silencing the lncRNA NORAD inhibits EMT of head and neck squamous cell carcinoma stem cells via miR‑26a‑5p. Mol Med Rep. 2021;24(5):743. 34435652 10.3892/mmr.2021.12383 PMC8430304

[129] Yuan S‐SF , Hung AC , Hsu C‐W , et al. CD44 mediates oral squamous cell carcinoma‐promoting activity of MRE11 via AKT signaling. J Pers Med. 2022;12(5):841. 35629265 10.3390/jpm12050841 PMC9144890

[130] Pastushenko I , Mauri F , Song Y , et al. Fat1 deletion promotes hybrid EMT state, tumour stemness and metastasis. Nature. 2021;589(7842):448‐455. 33328637 10.1038/s41586-020-03046-1 PMC7612440

[131] Sun X , Sun Y , Li J , et al. SOCS6 promotes radiosensitivity and decreases cancer cell stemness in esophageal squamous cell carcinoma by regulating c‐Kit ubiquitylation. Cancer Cell Int. 2021;21(1):1‐15. 33712005 10.1186/s12935-021-01859-2 PMC7953756

[132] Yokoyama S , Shigeishi H , Murodumi H , et al. TGF‐β1 induces amoeboid‐to‐mesenchymal transition of CD44high oral squamous cell carcinoma cells via miR‐422a downregulation through ERK activation and Cofilin‐1 phosphorylation. J Oral Pathol Med. 2021;50(2):155‐164. 33107637 10.1111/jop.13113

[133] Fernández‐Tabanera E , García‐García L , Rodríguez‐Martín C , et al. CD44 modulates cell migration and invasion in Ewing sarcoma cells. Int J Mol Sci. 2023;24(14):11774. 37511533 10.3390/ijms241411774 PMC10381016

[134] Fernández‐Tabanera E , Melero‐Fernández de Mera RM , Alonso J . CD44 in sarcomas: a comprehensive review and future perspectives. Front Oncol. 2022;12:909450. 35785191 10.3389/fonc.2022.909450 PMC9247467

[135] Skubitz KM , Wilson JD , Cheng EY , Lindgren BR , Boylan KLM , Skubitz APN . Effect of chemotherapy on cancer stem cells and tumor‐associated macrophages in a prospective study of preoperative chemotherapy in soft tissue sarcoma. J Transl Med. 2019;17(1):130. 30999901 10.1186/s12967-019-1883-6 PMC6471853

[136] Gerardo‐Ramírez M , Keggenhoff FL , Giam V , et al. CD44 contributes to the regulation of MDR1 protein and doxorubicin chemoresistance in osteosarcoma. Int J Mol Sci. 2022;23(15):8616. 35955749 10.3390/ijms23158616 PMC9368984

[137] Liu T , Yan Z , Liu Y , et al. CRISPR‐Cas9‐mediated silencing of CD44 in human highly metastatic osteosarcoma cells. Cell Physiol Biochem. 2018;46(3):1218‐1230. 29672299 10.1159/000489072

[138] Kogerman P , Sy M‐S , Culp LA . Counter‐selection for over‐expressed human CD44s in primary tumors versus lung metastases in a mouse fibrosarcoma model. Oncogene. 1997;15(12):1407‐1416. 9333016 10.1038/sj.onc.1201306

[139] Kuryu M , Ozaki T , Nishida K , Shibahara M , Kawai A , Inoue H . Expression of CD44 variants in osteosarcoma. J Cancer Res Clin Oncol. 1999;125(11):646‐652. 10541973 10.1007/s004320050329 PMC12169063

[140] Toole BP . Hyaluronan: from extracellular glue to pericellular cue. Nat Rev Cancer. 2004;4(7):528‐539. 15229478 10.1038/nrc1391

[141] Al‐Mansoob M , Gupta I , Stefan Rusyniak R , Ouhtit A . KYNU, a novel potential target that underpins CD44‐promoted breast tumour cell invasion. J Cell Mol Med. 2021;25(5):2309‐2314. 33486887 10.1111/jcmm.16296 PMC7933956

[142] Aruffo A , Stamenkovic I , Melnick M , Underhill CB , Seed B . CD44 is the principal cell surface receptor for hyaluronate. Cell. 1990;61(7):1303‐1313. 1694723 10.1016/0092-8674(90)90694-a

[143] Hua Q , Knudson CB , Knudson W . Internalization of hyaluronan by chondrocytes occurs via receptor‐mediated endocytosis. J Cell Sci. 1993;106(Pt 1):365‐375. 7505784 10.1242/jcs.106.1.365

[144] Soleymani M , Velashjerdi M , Shaterabadi Z , Barati A . One‐pot preparation of hyaluronic acid‐coated iron oxide nanoparticles for magnetic hyperthermia therapy and targeting CD44‐overexpressing cancer cells. Carbohydr Polym. 2020;237:116130. 32241421 10.1016/j.carbpol.2020.116130

[145] Bourguignon LY , Earle C , Shiina M . Activation of matrix hyaluronan‐mediated CD44 signaling, epigenetic regulation and chemoresistance in head and neck cancer stem cells. Int J Mol Sci. 2017;18(9):1849. 28837080 10.3390/ijms18091849 PMC5618498

[146] Golshani R , Lopez L , Estrella V , Kramer M , Iida N , Lokeshwar VB . Hyaluronic acid synthase‐1 expression regulates bladder cancer growth, invasion, and angiogenesis through CD44. Cancer Res. 2008;68(2):483‐491. 18199543 10.1158/0008-5472.CAN-07-2140

[147] Bourguignon LY , Xia W , Wong G . Hyaluronan‐mediated CD44 interaction with p300 and SIRT1 regulates β‐catenin signaling and NFκB‐specific transcription activity leading to MDR1 and Bcl‐xL gene expression and chemoresistance in breast tumor cells. J Biol Chem. 2009;284(5):2657‐2671. 19047049 10.1074/jbc.M806708200 PMC2631959

[148] Alamgeer M , Neil Watkins D , Banakh I , et al. A phase IIa study of HA‐irinotecan, formulation of hyaluronic acid and irinotecan targeting CD44 in extensive‐stage small cell lung cancer. Invest New Drugs. 2018;36(2):288‐298. 29277856 10.1007/s10637-017-0555-8

[149] Salari N , Mansouri K , Valipour E , et al. Hyaluronic acid‐based drug nanocarriers as a novel drug delivery system for cancer chemotherapy: a systematic review. DARU. 2021;29(2):439‐447. 34499323 10.1007/s40199-021-00416-6 PMC8602596

[150] Ahmed M , Sottnik JL , Dancik GM , et al. An osteopontin/CD44 axis in RhoGDI2‐mediated metastasis suppression. Cancer Cell. 2016;30(3):432‐443. 27593345 10.1016/j.ccell.2016.08.002 PMC5154333

[151] Ji J , Zheng S , Liu Y , et al. Increased expression of OPN contributes to idiopathic pulmonary fibrosis and indicates a poor prognosis. J Transl Med. 2023;21(1):640. 37726818 10.1186/s12967-023-04279-0 PMC10510122

[152] Pang X , Gong K , Zhang X , Wu S , Cui Y , Qian B‐Z . Osteopontin as a multifaceted driver of bone metastasis and drug resistance. Pharmacol Res. 2019;144:235‐244. 31028902 10.1016/j.phrs.2019.04.030

[153] Rao G , Wang H , Li B , et al. Reciprocal interactions between tumor‐associated macrophages and CD44‐positive cancer cells via osteopontin/CD44 promote tumorigenicity in colorectal cancer the interaction of OPN and CD44 in colorectal cancer. Clin Cancer Res. 2013;19(4):785‐797. 23251004 10.1158/1078-0432.CCR-12-2788

[154] Robertson BW , Bonsal L , Chellaiah MA . Regulation of Erk1/2 activation by osteopontin in PC3 human prostate cancer cells. Mol Cancer. 2010;9:260. 20868520 10.1186/1476-4598-9-260 PMC3098013

[155] Jiang Y‐J , Chao C‐C , Chang A‐C , et al. Cigarette smoke‐promoted increases in osteopontin expression attract mesenchymal stem cell recruitment and facilitate lung cancer metastasis. J Adv Res. 2022;41:77‐87. 36328755 10.1016/j.jare.2021.12.011 PMC9637482

[156] Klement JD , Paschall AV , Redd PS , et al. An osteopontin/CD44 immune checkpoint controls CD8+ T cell activation and tumor immune evasion. J Clin Invest. 2018;128(12):5549‐5560. 30395540 10.1172/JCI123360 PMC6264631

[157] Lu C , Liu Z , Klement JD , et al. WDR5‐H3K4me3 epigenetic axis regulates OPN expression to compensate PD‐L1 function to promote pancreatic cancer immune escape. J Immunother Cancer. 2021;9(7):e002624. 34326167 10.1136/jitc-2021-002624 PMC8323468

[158] Manou D , Karamanos NK , Theocharis AD . Tumorigenic functions of serglycin: Regulatory roles in epithelial to mesenchymal transition and oncogenic signaling. Semin Cancer Biol. 2020;62:108‐115. 31279836 10.1016/j.semcancer.2019.07.004

[159] Pejler G , Åbrink M , Wernersson S . Serglycin proteoglycan: regulating the storage and activities of hematopoietic proteases. Biofactors. 2009;35(1):61‐68. 19319847 10.1002/biof.11

[160] Guo J‐Y , Chiu C‐H , Wang M‐J , Li F‐A , Chen J‐Y . Proteoglycan serglycin promotes non‐small cell lung cancer cell migration through the interaction of its glycosaminoglycans with CD44. J Biomed Sci. 2020;27(1):1‐18. 31898491 10.1186/s12929-019-0600-3 PMC6939340

[161] Cao L , Luo FF , Huang HB , et al. The autoregulatory serglycin/CD44 axis drives stemness‐like phenotypes in TNBC in a β‐catenin‐dependent manner. Clin Transl Med. 2021;11(2):e311. 33634997 10.1002/ctm2.311 PMC7851355

[162] He Y , Cheng D , Lian C , et al. Serglycin induces osteoclastogenesis and promotes tumor growth in giant cell tumor of bone. Cell Death Dis. 2021;12(10):868. 34556636 10.1038/s41419-021-04161-1 PMC8460728

[163] Liang X , Liang X . Chondroitin sulfate modified and adriamycin preloaded hybrid nanoparticles for tumor‐targeted chemotherapy of lung cancer. Kaohsiung J Med Sci. 2021;37(5):411‐418. 33340254 10.1002/kjm2.12339 PMC11896459

[164] Lin WJ , Lee WC . Polysaccharide‐modified nanoparticles with intelligent CD44 receptor targeting ability for gene delivery. Int J Nanomed. 2018;13:3989. 10.2147/IJN.S163149 PMC6045904 30022822

[165] Tan T , Yang Q , Chen D , et al. Chondroitin sulfate‐mediated albumin corona nanoparticles for the treatment of breast cancer. Asian J Pharm Sci. 2021;16(4):508‐518. 34703499 10.1016/j.ajps.2021.03.004 PMC8520051

[166] Luo K , Xu F , Yao T , et al. TPGS and chondroitin sulfate dual‐modified lipid‐albumin nanosystem for targeted delivery of chemotherapeutic agent against multidrug‐resistant cancer. Int J Biol Macromol. 2021;183:1270‐1282. 34004196 10.1016/j.ijbiomac.2021.05.070

[167] Chu Y‐H , Liao W‐C , Ho Y‐J , Huang C‐H , Tseng T‐J , Liu C‐H . Targeting chondroitin sulfate reduces invasiveness of glioma cells by suppressing CD44 and integrin β1 expression. Cells. 2021;10(12):3594. 34944101 10.3390/cells10123594 PMC8700349

[168] Chrabańska M , Rynkiewicz M , Kiczmer P , Drozdzowska B . Immunohistochemical expression of CD44, MMP‐2, MMP‐9, and Ki‐67 as the prognostic markers in non‐clear cell renal cell carcinomas: a prospective cohort study. J Clin Med. 2022;11(17):5196. 36079127 10.3390/jcm11175196 PMC9457518

[169] Song X , Ding F , Luo W , et al. Knockdown of CD44 inhibits proliferation, migration, and invasiveness in hepatocellular carcinoma cells by modulating CXCR4/Wnt/β‐Catenin Axis. Acta Biochim Pol. 2023;70(1):117‐122. 36735564 10.18388/abp.2020_6319

[170] Lv Y , Zhao X , Zhu L , et al. Targeting intracellular MMPs efficiently inhibits tumor metastasis and angiogenesis. Theranostics. 2018;8(10):2830. 29774078 10.7150/thno.23209 PMC5957012

[171] Shi J , Ren Y , Ma J , et al. Novel CD44‐targeting and pH/redox‐dual‐stimuli‐responsive core–shell nanoparticles loading triptolide combats breast cancer growth and lung metastasis. J Nanobiotechnol. 2021;19(1):1‐22. 10.1186/s12951-021-00934-0 PMC8220850 34162396

[172] Ko YS , Jung EJ , Go S‐I , et al. Polyphenols extracted from Artemisia annua L. exhibit anti‐cancer effects on radio‐resistant MDA‐MB‐231 human breast cancer cells by suppressing stem cell phenotype, β‐catenin, and MMP‐9. Molecules. 2020;25(8):1916. 32326231 10.3390/molecules25081916 PMC7221914

[173] Lee Y‐M , Kim JM , Lee HJ , Seong I‐O , Kim K‐H . Immunohistochemical Expression of CD44, Matrix Metalloproteinase2 and Matrix Metalloproteinase9 in Renal Cell Carcinomas. Elsevier; 2019:742‐748. 10.1016/j.urolonc.2019.04.017 31053527

[174] Chen S‐Y , Jou I‐M , Ko P‐Y , et al. Amelioration of experimental tendinopathy by lentiviral CD44 gene therapy targeting senescence‐associated secretory phenotypes. Mol Ther Methods Clin Dev. 2022;26:157‐168. 35846572 10.1016/j.omtm.2022.06.006 PMC9254001

[175] Papadopoulou A , Kalodimou VE , Mavrogonatou E , et al. Decreased differentiation capacity and altered expression of extracellular matrix components in irradiation‐mediated senescent human breast adipose‐derived stem cells. IUBMB Life. 2022;74(10):969‐981. 35833571 10.1002/iub.2659

[176] Asano M , Tanaka S , Sakaguchi M . Effects of normothermic microwave irradiation on CD44+/CD24‒in breast cancer MDA‐MB‐231 and MCF‐7 cell lines. Biosci Biotechnol Biochem. 2020;84(1):103‐110. 31559912 10.1080/09168451.2019.1670044

[177] Nikitakis NG , Gkouveris I , Aseervatham J , Barahona K , Ogbureke KU . DSPP‐MMP20 gene silencing downregulates cancer stem cell markers in human oral cancer cells. Cell Mol Biol Lett. 2018;23(1):1‐14. 30002682 10.1186/s11658-018-0096-y PMC6040065

[178] Li L , Qi L , Qu T , et al. Epithelial splicing regulatory protein 1 inhibits the invasion and metastasis of lung adenocarcinoma. Am J Pathol. 2018;188(8):1882‐1894. 29803834 10.1016/j.ajpath.2018.04.012

[179] Senbanjo LT , AlJohani H , Majumdar S , Chellaiah MA . Characterization of CD44 intracellular domain interaction with RUNX2 in PC3 human prostate cancer cells. Cell Commun Signal. 2019;17(1):1‐13. 31331331 10.1186/s12964-019-0395-6 PMC6647163

[180] Kato H , Naiki‐Ito A , Yamada T , et al. The standard form of CD44 as a marker for invasion of encapsulated papillary carcinoma of the breast. Pathol Int. 2020;70(11):835‐843. 32783311 10.1111/pin.13001

[181] Sun X , Li K , Hase M , et al. Suppression of breast cancer‐associated bone loss with osteoblast proteomes via Hsp90ab1/moesin‐mediated inhibition of TGFβ/FN1/CD44 signaling. Theranostics. 2022;12(2):929‐943. 34976221 10.7150/thno.66148 PMC8692912

[182] Choi S , Yu J , Park A , et al. BMP‐4 enhances epithelial mesenchymal transition and cancer stem cell properties of breast cancer cells via Notch signaling. Sci Rep. 2019;9(1):11724. 31409851 10.1038/s41598-019-48190-5 PMC6692307

[183] Frahs SM , Reeck JC , Yocham KM , et al. Prechondrogenic ATDC5 cell attachment and differentiation on graphene foam; modulation by surface functionalization with fibronectin. ACS Appl Mater Interfaces. 2019;11(45):41906‐41924. 31639302 10.1021/acsami.9b14670 PMC6858527

[184] Hu C , Li M , Guo T , et al. Anti‐metastasis activity of curcumin against breast cancer via the inhibition of stem cell‐like properties and EMT. Phytomedicine. 2019;58:152740. 31005718 10.1016/j.phymed.2018.11.001

[185] Li N , Wang C , Sun S , et al. Microgravity‐induced alterations of inflammation‐related mechanotransduction in endothelial cells on Board SJ‐10 satellite. Front Physiol. 2018;9:1025. 30108515 10.3389/fphys.2018.01025 PMC6079262

[186] Huang G‐X , Qi M‐F , Li X‐L , Tang F , Zhu L . Involvement of upregulation of fibronectin in the pro‑adhesive and pro‑survival effects of glucocorticoid on melanoma cells. Mol Med Rep. 2018;17(2):3380‐3387. 29257300 10.3892/mmr.2017.8269

[187] Hong Y , Qin H , Li Y , et al. FNDC3B circular RNA promotes the migration and invasion of gastric cancer cells via the regulation of E‐cadherin and CD44 expression. J Cell Physiol. 2019;234(11):19895‐19910. 30963578 10.1002/jcp.28588 PMC6766960

[188] Figiel I , Kruk PK , Zaręba‐Kozioł M , et al. MMP‐9 signaling pathways that engage Rho GTPases in brain plasticity. Cells. 2021;10(1):166. 33467671 10.3390/cells10010166 PMC7830260

[189] Tata P , Gondaliya P , Sunkaria A , Srivastava A , Kalia K . Modulation of CD44, EGFR and RAC pathway genes (WAVE complex) in epithelial cancers. Curr Pharm Des. 2019;25(8):833‐848. 30799784 10.2174/1381612825666190222143044

[190] Bai R‐J , Liu D , Li Y‐S , et al. OPN inhibits autophagy through CD44, integrin and the MAPK pathway in osteoarthritic chondrocytes. Front Endocrinol (Lausanne). 2022;13:919366. 36034459 10.3389/fendo.2022.919366 PMC9411521

[191] Bourguignon LYW , Earle C , Wong G , Spevak CC , Krueger K . Stem cell marker (Nanog) and Stat‐3 signaling promote MicroRNA‐21 expression and chemoresistance in hyaluronan/CD44‐activated head and neck squamous cell carcinoma cells. Oncogene. 2012;31(2):149‐160. 21685938 10.1038/onc.2011.222 PMC3179812

[192] Medrano‐González PA , Rivera‐Ramírez O , Montaño LF , Rendón‐Huerta EP . Proteolytic processing of CD44 and its implications in cancer. Stem Cells Int. 2021;2021:6667735. 33505471 10.1155/2021/6667735 PMC7811561

[193] Primeaux M , Gowrikumar S , Dhawan P . Role of CD44 isoforms in epithelial‐mesenchymal plasticity and metastasis. Clin Exp Metastasis. 2022;39(3):391‐406. 35023031 10.1007/s10585-022-10146-x PMC10042269

[194] Roy R , Mandal S , Chakrabarti J , Saha P , Panda CK . Downregulation of hyaluronic acid‐CD44 signaling pathway in cervical cancer cell by natural polyphenols Plumbagin, Pongapin and Karanjin. Mol Cell Biochem. 2021;476(10):3701‐3709. 34081254 10.1007/s11010-021-04195-1

[195] Jiang P , Li F , Liu Z , Hao S , Gao J , Li S . BTB and CNC homology 1 (Bach1) induces lung cancer stem cell phenotypes by stimulating CD44 expression. Respir Res. 2021;22(1):320. 34949193 10.1186/s12931-021-01918-2 PMC8697453

[196] Petri BJ , Piell KM , Whitt GCS , et al. HNRNPA2B1 regulates tamoxifen‐and fulvestrant‐sensitivity and hallmarks of endocrine resistance in breast cancer cells. Cancer Lett. 2021;518:152‐168. 34273466 10.1016/j.canlet.2021.07.015 PMC8358706

[197] Erdogan S , Turkekul K . Neferine inhibits proliferation and migration of human prostate cancer stem cells through p38 MAPK/JNK activation. J Food Biochem. 2020;44(7):e13253. 32394497 10.1111/jfbc.13253

[198] Lin C , Yuan H , Wang W , et al. Importance of PNO1 for growth and survival of urinary bladder carcinoma: role in core‐regulatory circuitry. J Cell Mol Med. 2020;24(2):1504‐1515. 31800162 10.1111/jcmm.14835 PMC6991670

[199] Guo Q , Liu Y , He Y , et al. CD44 activation state regulated by the CD44v10 isoform determines breast cancer proliferation. Oncol Rep. 2021;45(4):7. 33649828 10.3892/or.2021.7958 PMC7876991

[200] Erdogan S , Turkekul K , Dibirdik I , Doganlar ZB , Doganlar O , Bilir A . Midkine silencing enhances the anti‐prostate cancer stem cell activity of the flavone apigenin: cooperation on signaling pathways regulated by ERK, p38, PTEN, PARP, and NF‐κB. Invest New Drugs. 2020;38(2):246‐263. 30993586 10.1007/s10637-019-00774-8

[201] Jang T‐H , Huang W‐C , Tung S‐L , et al. MicroRNA‐485‐5p targets keratin 17 to regulate oral cancer stemness and chemoresistance via the integrin/FAK/Src/ERK/β‐catenin pathway. J Biomed Sci. 2022;29(1):42. 35706019 10.1186/s12929-022-00824-z PMC9202219

[202] Sun J , Xu Y , Liu J , Cui H , Cao H , Ren J . PDRG1 promotes the proliferation and migration of GBM cells by the MEK/ERK/CD44 pathway. Cancer Sci. 2022;113(2):500‐516. 34812552 10.1111/cas.15214 PMC8819344

[203] Zhao Y , Kang J‐H , Yoo K‐C , Kang S‐G , Lee H‐J , Lee S‐J . K‐RAS acts as a critical regulator of CD44 to promote the invasiveness and stemness of GBM in response to ionizing radiation. Int J Mol Sci. 2021;22(20):10923. 34681583 10.3390/ijms222010923 PMC8539357

[204] Wang Y‐Y , Vadhan A , Chen P‐H , et al. Cd44 promotes lung cancer cell metastasis through erk–zeb1 signaling. Cancers (Basel). 2021;13(16):4057. 34439211 10.3390/cancers13164057 PMC8392539

[205] Thirusangu P , Ray U , Sarkar Bhattacharya S , et al. PFKFB3 regulates cancer stemness through the hippo pathway in small cell lung carcinoma. Oncogene. 2022;41(33):4003‐4017. 35804016 10.1038/s41388-022-02391-x PMC9374593

[206] Zhang J , He X , Wan Y , et al. CD44 promotes hepatocellular carcinoma progression via upregulation of YAP. Exp Hematol Oncol. 2021;10(1):54. 34798909 10.1186/s40164-021-00247-w PMC8603576

[207] Giraud J , Molina‐Castro S , Seeneevassen L , et al. Verteporfin targeting YAP1/TAZ‐TEAD transcriptional activity inhibits the tumorigenic properties of gastric cancer stem cells. Int J Cancer. 2020;146(8):2255‐2267. 31489619 10.1002/ijc.32667

[208] Liu Y , Song Y , Cao M , et al. A novel EHD1/CD44/Hippo/SP1 positive feedback loop potentiates stemness and metastasis in lung adenocarcinoma. Clin Transl Med. 2022;12(4):e836. 35485206 10.1002/ctm2.836 PMC9786223

[209] Tanaka K , Osada H , Murakami‐Tonami Y , Horio Y , Hida T , Sekido Y . Statin suppresses Hippo pathway‐inactivated malignant mesothelioma cells and blocks the YAP/CD44 growth stimulatory axis. Cancer Lett. 2017;385:215‐224. 27773750 10.1016/j.canlet.2016.10.020

[210] Lai C‐J , Lin C‐Y , Liao W‐Y , Hour T‐C , Wang H‐D , Chuu C‐P . CD44 promotes migration and invasion of docetaxel‐resistant prostate cancer cells likely via induction of hippo‐yap signaling. Cells. 2019;8(4):295. 30935014 10.3390/cells8040295 PMC6523775

[211] Zhao N , Zhou L , Lu Q , et al. SOX2 maintains the stemness of retinoblastoma stem‐like cells through Hippo/YAP signaling pathway. Exp Eye Res. 2022;214:108887. 34890603 10.1016/j.exer.2021.108887

[212] Zhou Y , Qiu S , Kim JT , et al. Garcinone C suppresses tumorsphere formation and invasiveness by hedgehog/Gli1 signaling in colorectal cancer stem‐like cells. J Agric Food Chem. 2022;70(26):7941‐7952. 35749593 10.1021/acs.jafc.2c01891

[213] Lall SP , Alsafwani ZW , Batra SK , Seshacharyulu P . ASPORIN: A root of the matter in tumors and their host environment. Biochim Biophys Acta Rev Cancer. 2023;1879(1):189029. 38008263 10.1016/j.bbcan.2023.189029 PMC10872503

[214] Huang JL , Oshi M , Endo I , Takabe K . Clinical relevance of stem cell surface markers CD133, CD24, and CD44 in colorectal cancer. Am J Cancer Res. 2021;11(10):5141‐5154. 34765317 PMC8569346

[215] Li C , Du Y , Yang Z , et al. GALNT1‐mediated glycosylation and activation of sonic hedgehog signaling maintains the self‐renewal and tumor‐initiating capacity of bladder cancer stem cells. Cancer Res. 2016;76(5):1273‐1283. 26676748 10.1158/0008-5472.CAN-15-2309

[216] Tsao A‐N , Chuang Y‐S , Lin Y‐C , Su Y , Chao T‐C . Dinaciclib inhibits the stemness of two subtypes of human breast cancer cells by targeting the FoxM1 and Hedgehog signaling pathway. Oncol Rep. 2022;47(5):105. 35417031 10.3892/or.2022.8316

[217] Zhou D , He Y , Li H , Huang W . KLK6 mediates stemness and metabolism of gastric carcinoma cells via the PI3K/AKT/mTOR signaling pathway. Oncol Lett. 2021;22(6):824. 34691251 10.3892/ol.2021.13085 PMC8527834

[218] Fan L , Peng C , Zhu X , et al. Dihydrotanshinone I enhances cell adhesion and inhibits cell migration in osteosarcoma U‐2 OS cells through CD44 and chemokine signaling. Molecules. 2022;27(12):3714. 35744840 10.3390/molecules27123714 PMC9231138

[219] Coleman K‐L , Chiaramonti M , Haddad B , et al. Phosphorylation of IGFBP‐3 by casein kinase 2 blocks its interaction with hyaluronan, enabling HA‐CD44 signaling leading to increased NSCLC cell survival and cisplatin resistance. Cells. 2023;12(3):405. 36766747 10.3390/cells12030405 PMC9913475

[220] Thanee M , Dokduang H , Kittirat Y , et al. CD44 modulates metabolic pathways and altered ROS‐mediated Akt signal promoting cholangiocarcinoma progression. PLoS One. 2021;16(3):e0245871. 33780455 10.1371/journal.pone.0245871 PMC8007026

[221] Geng B , Pan J , Zhao T , et al. Chitinase 3‐like 208‐CD44 interaction promotes metastasis and epithelial‐to‐mesenchymal transition through β‐catenin/Erk/Akt signaling in gastric cancer. J Exp Clin Cancer Res. 2018;37(1):1‐20. 30165890 10.1186/s13046-018-0876-2 PMC6117920

[222] Xie P , Yan J , Wu M , et al. CD44 potentiates hepatocellular carcinoma migration and extrahepatic metastases via the AKT/ERK signaling CXCR4 axis. Ann Transl Med. 2022;10(12):689. 35845518 10.21037/atm-22-2482 PMC9279758

[223] Kashyap T , Pramanik KK , Nath N , et al. Crosstalk between Raf‐MEK‐ERK and PI3K‐Akt‐GSK3β signaling networks promotes chemoresistance, invasion/migration and stemness via expression of CD44 variants (v4 and v6) in oral cancer. Oral Oncol. 2018;86:234‐243. 30409306 10.1016/j.oraloncology.2018.09.028

[224] Wang J , Li X , Wu H , et al. EMP1 regulates cell proliferation, migration, and stemness in gliomas through PI3K‐AKT signaling and CD44. J Cell Biochem. 2019;120(10):17142‐17150. 31111534 10.1002/jcb.28974

[225] Koh YW , Han J‐H , Haam S . Expression of PD‐L1, cancer stem cell and epithelial‐mesenchymal transition phenotype in non‐small cell lung cancer. Pathology (Phila). 2021;53(2):239‐246. 10.1016/j.pathol.2020.07.009 33036771

[226] Chen J‐T , Hsu Y‐L , Hsu Y‐C , et al. Id2 exerts tumor suppressor properties in lung cancer through its effects on cancer cell invasion and migration. Front Oncol. 2022;12:801300. 35982951 10.3389/fonc.2022.801300 PMC9379288

[227] Su J , Wu S , Wu H , Li L , Guo T . CD44 is functionally crucial for driving lung cancer stem cells metastasis through Wnt/β‐catenin‐FoxM1‐Twist signaling. Mol Carcinog. 2016;55(12):1962‐1973. 26621583 10.1002/mc.22443

[228] Acikgoz E , Tatar C , Oktem G . Triptolide inhibits CD133+ /CD44+ colon cancer stem cell growth and migration through triggering apoptosis and represses epithelial‐mesenchymal transition via downregulating expressions of snail, slug, and twist. J Cell Biochem. 2020;121(5‐6):3313‐3324. 31904143 10.1002/jcb.29602

[229] Mahmoudian RA , Gharaie ML , Abbaszadegan MR , et al. Crosstalk between MMP‐13, CD44, and TWIST1 and its role in regulation of EMT in patients with esophageal squamous cell carcinoma. Mol Cell Biochem. 2021;476(6):2465‐2478. 33604811 10.1007/s11010-021-04089-2

[230] Heydari M , Hosseinzadeh Colagar A , Moudi E . Mutant Allele of CD44 (rs8193C>T) and Pum2 regulatory element as a prognosis factor of prostate neoplasms: a case‐control and in silico studies. Cell J. 2022;24(12):723‐731. 36527344 10.22074/cellj.2022.8468 PMC9790067

[231] Byun J‐Y , Huang K , Lee JS , et al. Targeting HIF‐1α/NOTCH1 pathway eliminates CD44+ cancer stem‐like cell phenotypes, malignancy, and resistance to therapy in head and neck squamous cell carcinoma. Oncogene. 2022;41(9):1352‐1363. 35013621 10.1038/s41388-021-02166-w

[232] Bai J , Chen W‐B , Zhang X‐Y , et al. HIF‐2α regulates CD44 to promote cancer stem cell activation in triple‐negative breast cancer via PI3K/AKT/mTOR signaling. World J Stem Cells. 2020;12(1):87‐99. 32110277 10.4252/wjsc.v12.i1.87 PMC7031759

[233] Gu X , Zhang J , Shi Y , et al. ESM1/HIF‑1α pathway modulates chronic intermittent hypoxia‑induced non‑small‑cell lung cancer proliferation, stemness and epithelial‑mesenchymal transition. Oncol Rep. 2021;45(3):1226‐1234. 33650648 10.3892/or.2020.7913

[234] Inoue A , Ohnishi T , Nishikawa M , et al. A narrative review on CD44's role in glioblastoma invasion, proliferation, and tumor recurrence. Cancers (Basel). 2023;15(19):4898. 37835592 10.3390/cancers15194898 PMC10572085

[235] Johansson E , Grassi ES , Pantazopoulou V , et al. CD44 Interacts with HIF‐2α to modulate the hypoxic phenotype of perinecrotic and perivascular glioma cells. Cell Rep. 2017;20(7):1641‐1653. 28813675 10.1016/j.celrep.2017.07.049

[236] Yang H‐L , Lin P‐Y , Vadivalagan C , Lin Y‐A , Lin K‐Y , Hseu Y‐C . Coenzyme Q0 defeats NLRP3‐mediated inflammation, EMT/metastasis, and Warburg effects by inhibiting HIF‐1α expression in human triple‐negative breast cancer cells. Arch Toxicol. 2023;97(4):1047‐1068. 36847822 10.1007/s00204-023-03456-w

[237] Piao L , Li H , Feng Y , Yang Z , Kim S , Xuan Y . SET domain‐containing 5 is a potential prognostic biomarker that promotes esophageal squamous cell carcinoma stemness. Exp Cell Res. 2020;389(1):111861. 31981592 10.1016/j.yexcr.2020.111861

[238] Liang G , Li S , Du W , Ke Q , Cai J , Yang J . Hypoxia regulates CD44 expression via hypoxia‐inducible factor‐1α in human gastric cancer cells. Oncol Lett. 2017;13(2):967‐972. 28356986 10.3892/ol.2016.5473 PMC5351347

[239] Nam K , Oh S , Shin I . Ablation of CD44 induces glycolysis‐to‐oxidative phosphorylation transition via modulation of the c‐Src‐Akt‐LKB1‐AMPKα pathway. Biochem J. 2016;473(19):3013‐3030. 27458252 10.1042/BCJ20160613

[240] Kim S , Cho CY , Lee D , et al. CD133‐induced TM4SF5 expression promotes sphere growth via recruitment and blocking of protein tyrosine phosphatase receptor type F (PTPRF). Cancer Lett. 2018;438:219‐231. 30217560 10.1016/j.canlet.2018.09.009

[241] Liu J , Chen X , Ward T , et al. Niclosamide inhibits epithelial‐mesenchymal transition and tumor growth in lapatinib‐resistant human epidermal growth factor receptor 2‐positive breast cancer. Int J Biochem Cell Biol. 2016;71:12‐23. 26643609 10.1016/j.biocel.2015.11.014

[242] Nam K , Oh S , Lee K‐M , Yoo S‐A , Shin I . CD44 regulates cell proliferation, migration, and invasion via modulation of c‐Src transcription in human breast cancer cells. Cell Signal. 2015;27(9):1882‐1894. 25979842 10.1016/j.cellsig.2015.05.002

[243] Bourguignon LYW , Wong G , Earle C , Krueger K , Spevak CC . Hyaluronan‐CD44 interaction promotes c‐Src‐mediated twist signaling, microRNA‐10b expression, and RhoA/RhoC up‐regulation, leading to Rho‐kinase‐associated cytoskeleton activation and breast tumor cell invasion. J Biol Chem. 2010;285(47):36721‐36735. 20843787 10.1074/jbc.M110.162305 PMC2978601

[244] Lee D , Na J , Ryu J , et al. Interaction of tetraspan (in) TM4SF5 with CD44 promotes self‐renewal and circulating capacities of hepatocarcinoma cells. Hepatology. 2015;61(6):1978‐1997. 25627085 10.1002/hep.27721

[245] Katoh M . Multi‑layered prevention and treatment of chronic inflammation, organ fibrosis and cancer associated with canonical WNT/β‑catenin signaling activation (Review). Int J Mol Med. 2018;42(2):713‐725. 29786110 10.3892/ijmm.2018.3689 PMC6034925

[246] Roy S , Kar M , Roy S , et al. Inhibition of CD44 sensitizes cisplatin‐resistance and affects Wnt/β‐catenin signaling in HNSCC cells. Int J Biol Macromol. 2020;149:501‐512. 31953176 10.1016/j.ijbiomac.2020.01.131

[247] Guo K , Duan J , Lu J , et al. Tumor necrosis factor‐α‐inducing protein of Helicobacter pylori promotes epithelial‐mesenchymal transition and cancer stem‐like cells properties via activation of Wnt/β‐catenin signaling pathway in gastric cancer cells. Pathog Dis. 2022;80(1):ftac025. 35776950 10.1093/femspd/ftac025

[248] Zhang M , Wang X , Xia X , Fang X , Zhang T , Huang F . Endometrial epithelial cells‐derived exosomes deliver microRNA‐30c to block the BCL9/Wnt/CD44 signaling and inhibit cell invasion and migration in ovarian endometriosis. Cell Death Discov. 2022;8(1):151. 35368023 10.1038/s41420-022-00941-6 PMC8976844

[249] Li Q , Li Y , Jiang H , et al. Vitamin D suppressed gastric cancer cell growth through downregulating CD44 expression in vitro and in vivo. Nutrition. 2021;91:111413. 34450383 10.1016/j.nut.2021.111413

[250] Fan Y , Cheng H , Liu Y , et al. Metformin anticancer: Reverses tumor hypoxia induced by bevacizumab and reduces the expression of cancer stem cell markers CD44/CD117 in human ovarian cancer SKOV3 cells. Front Pharmacol. 2022;13:955984. 36046821 10.3389/fphar.2022.955984 PMC9421358

[251] Kim M , Lee JS , Kim W , et al. Aptamer‐conjugated nano‐liposome for immunogenic chemotherapy with reversal of immunosuppression. J Control Release. 2022;348:893‐910. 35760233 10.1016/j.jconrel.2022.06.039

[252] Espejo‐Román JM , Rubio‐Ruiz B , Cano‐Cortés V , et al. Selective anticancer therapy based on a HA‐CD44 interaction inhibitor loaded on polymeric nanoparticles. Pharmaceutics. 2022;14(4):788. 35456622 10.3390/pharmaceutics14040788 PMC9032636

[253] Chen Y , Wang H , Zuo Y , Li N , Ding M , Li C . A novel monoclonal antibody KMP1 has potential antitumor activity of bladder cancer by blocking CD44 in vivo and in vitro. Cancer Med. 2018;7(5):2064‐2077. 29577645 10.1002/cam4.1446 PMC5943472

[254] Takei J , Kaneko MK , Ohishi T , et al. A defucosylated anti‑CD44 monoclonal antibody 5‑mG2a‑f exerts antitumor effects in mouse xenograft models of oral squamous cell carcinoma. Oncol Rep. 2020;44(5):1949‐1960. 33000243 10.3892/or.2020.7735 PMC7550977

[255] Khan F , Gurung S , Gunassekaran GR , et al. Identification of novel CD44v6‐binding peptides that block CD44v6 and deliver a pro‐apoptotic peptide to tumors to inhibit tumor growth and metastasis in mice. Theranostics. 2021;11(3):1326‐1344. 33391537 10.7150/thno.50564 PMC7738880

[256] Price D , Muterspaugh R , Clegg B , et al. IGFBP‐3 blocks hyaluronan‐CD44 signaling, leading to increased acetylcholinesterase levels in A549 cell media and apoptosis in a p53‐dependent manner. Sci Rep. 2020;10(1):5083. 32193421 10.1038/s41598-020-61743-3 PMC7081274

[257] Zheng J , Zhao S , Yu X , Huang S , Liu HY . Simultaneous targeting of CD44 and EpCAM with a bispecific aptamer effectively inhibits intraperitoneal ovarian cancer growth. Theranostics. 2017;7(5):1373‐1388. 28435472 10.7150/thno.17826 PMC5399600

[258] Wang H , Zhu Z , Zhang G , et al. AS1411 aptamer/hyaluronic acid‐bifunctionalized microemulsion co‐loading shikonin and docetaxel for enhanced antiglioma therapy. J Pharm Sci. 2019;108(11):3684‐3694. 31465736 10.1016/j.xphs.2019.08.017

[259] Zhu Y , Fu F , Wang Z , et al. Polyphyllin VII is a potential drug targeting CD44 positive colon cancer cells. Curr Cancer Drug Targets. 2022;22(5):426‐435. 35249490 10.2174/1568009622666220304110222

[260] Li J , Li M , Tian L , et al. Facile strategy by hyaluronic acid functional carbon dot‐doxorubicin nanoparticles for CD44 targeted drug delivery and enhanced breast cancer therapy. Int J Pharm. 2020;578:119122. 32035259 10.1016/j.ijpharm.2020.119122

[261] Yilmaz Ç , Köksoy S , Çeker T , Aslan M . Diclofenac down‐regulates COX‐2 induced expression of CD44 and ICAM‐1 in human HT29 colorectal cancer cells. Naunyn Schmiedebergs Arch Pharmacol. 2021;394(11):2259‐2272. 34436652 10.1007/s00210-021-02139-6

[262] Chen D , Li D , Xu X‐B , et al. Galangin inhibits epithelial‐mesenchymal transition and angiogenesis by downregulating CD44 in glioma. J Cancer. 2019;10(19):4499‐4508. 31528214 10.7150/jca.31487 PMC6746128

[263] Jobani BM , Najafzadeh N , Mazani M , Arzanlou M , Vardin MM . Molecular mechanism and cytotoxicity of allicin and all‐trans retinoic acid against CD44+ versus CD117+ melanoma cells. Phytomedicine. 2018;48:161‐169. 30195874 10.1016/j.phymed.2018.05.013

[264] Lee H‐J , Lim SM , Jang HY , Kim YR , Hong J‐S , Kim GJ . miR‐373‐3p regulates invasion and migration abilities of trophoblast cells via targeted CD44 and radixin. Int J Mol Sci. 2021;22(12):6260. 34200891 10.3390/ijms22126260 PMC8230484

[265] Gao Z , Ye X , Bordeaux A , et al. miR‐26b regulates cell proliferation and apoptosis of CD117+CD44+ ovarian cancer stem cells by targeting PTEN. Eur J Histochem. 2021;65(1):3186. 33634678 10.4081/ejh.2021.3186 PMC7883108

[266] Li WJ , Wang Y , Liu R , et al. MicroRNA‐34a: potent tumor suppressor, cancer stem cell inhibitor, and potential anticancer therapeutic. Front Cell Dev Biol. 2021;9:640587. 33763422 10.3389/fcell.2021.640587 PMC7982597

[267] Yu Y , Nangia‐Makker P , Farhana L , Rajendra SG , Levi E , Majumdar APN . miR‐21 and miR‐145 cooperation in regulation of colon cancer stem cells. Mol Cancer. 2015;14:98. 25928322 10.1186/s12943-015-0372-7 PMC4415383

[268] Liu C , Kelnar K , Liu B , et al. The microRNA miR‐34a inhibits prostate cancer stem cells and metastasis by directly repressing CD44. Nat Med. 2011;17(2):211‐215. 21240262 10.1038/nm.2284 PMC3076220

[269] Feng S , Wang K , Shao Z , Lin Q , Li B , Liu P . MiR‐373/miR‐520s‐CD44 axis significantly inhibits the growth and invasion of human glioblastoma cells. Arch Med Res. 2022;53(6):550‐561. 36115716 10.1016/j.arcmed.2022.08.003

[270] Li H‐N , Zhang H‐M , Li X‐R , et al. MiR‐205‐5p/GGCT attenuates growth and metastasis of papillary thyroid cancer by regulating CD44. Endocrinology. 2022;163(4):bqac022. 35213720 10.1210/endocr/bqac022 PMC8944316

[271] Yao H , Sun L , Li J , et al. A novel therapeutic siRNA nanoparticle designed for dual‐targeting CD44 and Gli1 of gastric cancer stem cells. Int J Nanomed. 2020;15:7013‐7034. 10.2147/IJN.S260163 PMC7522319 33061365

[272] Mahinfar P , Mokhtarzadeh A , Baradaran B , Siasi Torbati E . Antiproliferative activity of CD44 siRNA‐PEI‐PEG nanoparticles in glioblastoma: involvement of AKT signaling. Res Pharm Sci. 2022;17(1):78‐85. 34909046 10.4103/1735-5362.329928 PMC8621842

[273] Alemohammad H , Motafakkerazad R , Asadzadeh Z , et al. siRNA‐mediated silencing of Nanog reduces stemness properties and increases the sensitivity of HepG2 cells to cisplatin. Gene. 2022;821:146333. 35182674 10.1016/j.gene.2022.146333

[274] Zou W , Zhang Y , Bai G , et al. siRNA‐induced CD44 knockdown suppresses the proliferation and invasion of colorectal cancer stem cells through inhibiting epithelial‐mesenchymal transition. J Cell Mol Med. 2022;26(7):1969‐1978. 35229451 10.1111/jcmm.17221 PMC8980945

[275] Vahidian F , Safarzadeh E , Mohammadi A , et al. siRNA‐mediated silencing of CD44 delivered by Jet Pei enhanced Doxorubicin chemo sensitivity and altered miRNA expression in human breast cancer cell line (MDA‐MB468). Mol Biol Rep. 2020;47(12):9541‐9551. 33206362 10.1007/s11033-020-05952-z

[276] Yin J , Zhang H , Wu X , et al. CD44 inhibition attenuates EGFR signaling and enhances cisplatin sensitivity in human EGFR wild‑type non‑small‑cell lung cancer cells. Int J Mol Med. 2020;45(6):1783‐1792. 32236608 10.3892/ijmm.2020.4562 PMC7169661

[277] Porcellini S , Asperti C , Corna S , et al. CAR T cells redirected to CD44v6 control tumor growth in lung and ovary adenocarcinoma bearing mice. Front Immunol. 2020;11:99. 32117253 10.3389/fimmu.2020.00099 PMC7010926

[278] Cao Y , Efetov SK , He M , et al. Updated clinical perspectives and challenges of chimeric antigen receptor‐T cell therapy in colorectal cancer and invasive breast cancer. Arch Immunol Ther Exp (Warsz). 2023;71(1):19. 37566162 10.1007/s00005-023-00684-x

[279] Grunewald CM , Haist C , König C , et al. Epigenetic priming of bladder cancer cells with decitabine increases cytotoxicity of human EGFR and CD44v6 CAR engineered T‐cells. Front Immunol. 2021;12:782448. 34868059 10.3389/fimmu.2021.782448 PMC8637820

[280] Tang L , Huang H , Tang Y , et al. CD44v6 chimeric antigen receptor T cell specificity towards AML with FLT3 or DNMT3A mutations. Clin Transl Med. 2022;12(9):e1043. 36163632 10.1002/ctm2.1043 PMC9513046

[281] Haist C , Schulte E , Bartels N , et al. CD44v6‐targeted CAR T‐cells specifically eliminate CD44 isoform 6 expressing head/neck squamous cell carcinoma cells. Oral Oncol. 2021;116:105259. 33895463 10.1016/j.oraloncology.2021.105259

[282] Wang H , Ye X , Ju Y , et al. Minicircle DNA‐mediated CAR T cells targeting CD44 suppressed hepatocellular carcinoma both in vitro and in vivo. Onco Targets Ther. 2020;13:3703‐3716. 32440140 10.2147/OTT.S247836 PMC7210041

[283] Xia H , Hao M , Li K , et al. CD44 and HAP‐conjugated hADSCs as living materials for targeted tumor therapy and bone regeneration. Adv Sci (Weinh). 2023;10(20):e2206393. 37156753 10.1002/advs.202206393 PMC10369264

[284] Gonzalez‐Valdivieso J , Vallejo R , Rodriguez‐Rojo S , et al. CD44‐targeted nanoparticles for co‐delivery of docetaxel and an Akt inhibitor against colorectal cancer. Biomater Adv. 2023;154:213595. 37639856 10.1016/j.bioadv.2023.213595

[285] Uma Maheswari RT , Ajithkumar V , Varalakshmi P , Rajan M . CD44 tagged hyaluronic acid ‐ chitosan liposome carrier for the delivery of berberine and doxorubicin into lung cancer cells. Int J Biol Macromol. 2023;253(Pt 2):126599. 37652327 10.1016/j.ijbiomac.2023.126599

[286] Liu P , Chen N , Yan L , et al. Preparation, characterisation and in vitro and in vivo evaluation of CD44‐targeted chondroitin sulphate‐conjugated doxorubicin PLGA nanoparticles. Carbohydr Polym. 2019;213:17‐26. 30879657 10.1016/j.carbpol.2019.02.084

[287] Byeon Y , Lee J‐W , Choi WS , et al. CD44‐targeting PLGA nanoparticles incorporating paclitaxel and FAK siRNA overcome chemoresistance in epithelial ovarian cancer overcoming chemoresistance by HA‐PLGA‐NP in ovarian cancer. Cancer Res. 2018;78(21):6247‐6256. 30115698 10.1158/0008-5472.CAN-17-3871

[288] Seok H‐Y , Rejinold NS , Lekshmi KM , Cherukula K , Park I‐K , Kim Y‐C . CD44 targeting biocompatible and biodegradable hyaluronic acid cross‐linked zein nanogels for curcumin delivery to cancer cells: in vitro and in vivo evaluation. J Control Release. 2018;280:20‐30. 29723613 10.1016/j.jconrel.2018.04.050

[289] Liang Y , Wang Y , Wang L , et al. Self‐crosslinkable chitosan‐hyaluronic acid dialdehyde nanoparticles for CD44‐targeted siRNA delivery to treat bladder caner. Bioact Mater. 2021;6(2):433‐446. 32995671 10.1016/j.bioactmat.2020.08.019 PMC7490593

[290] Wang Y , Guo Y , Lin H , et al. Expression of CD44 in tumor tissue and serum of small cell lung cancer and its clinical prognostic significance. Zhongguo Fei Ai Za Zhi. 2021;24(8):583‐590. 34187156 10.3779/j.issn.1009-3419.2021.104.10 PMC8387646

[291] Agrawal A , Datta C , Panda CK , Pal DK . Association of beta‐catenin and CD44 in the development of renal cell carcinoma. Urologia. 2021;88(2):125‐129. 33300451 10.1177/0391560320980672

[292] Menke‐van der Houven van Oordt CW , Gomez‐Roca C , van Herpen C , et al. First‐in‐human phase I clinical trial of RG7356, an anti‐CD44 humanized antibody, in patients with advanced, CD44‐expressing solid tumors. Oncotarget. 2016;7(48):80046‐80058. 27507056 10.18632/oncotarget.11098 PMC5346770

[293] Gong L , Zhou H , Zhang S , et al. CD44‐targeting drug delivery system of exosomes loading forsythiaside a combats liver fibrosis via regulating NLRP3‐mediated pyroptosis. Adv Healthc Mater. 2023;12(11):e2202228. 36603210 10.1002/adhm.202202228

[294] Schonberg DL , Miller TE , Wu Q , et al. Preferential iron trafficking characterizes glioblastoma stem‐like cells. Cancer Cell. 2015;28(4):441‐455. 26461092 10.1016/j.ccell.2015.09.002 PMC4646058

[295] Mace TA , Shakya R , Pitarresi JR , et al. IL‐6 and PD‐L1 antibody blockade combination therapy reduces tumour progression in murine models of pancreatic cancer. Gut. 2018;67(2):320‐332. 27797936 10.1136/gutjnl-2016-311585 PMC5406266

[296] Nanbu T , Umemura N , Ohkoshi E , Nanbu K , Sakagami H , Shimada J . Combined SN‐38 and gefitinib treatment promotes CD44 degradation in head and neck squamous cell carcinoma cells. Oncol Rep. 2018;39(1):367‐375. 29192320 10.3892/or.2017.6105

